# Depolymerization within a Circular Plastics System

**DOI:** 10.1021/acs.chemrev.3c00739

**Published:** 2024-02-22

**Authors:** Robbie A. Clark, Michael P. Shaver

**Affiliations:** † Department of Materials, School of Natural Sciences, University of Manchester, Manchester M13 9PL, United Kingdom; ‡ Sustainable Materials Innovation Hub, Henry Royce Institute, University of Manchester, Manchester M13 9PL, United Kingdom

## Abstract

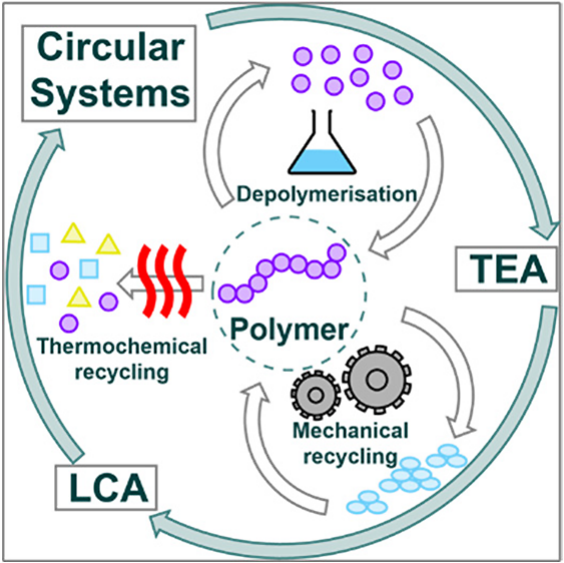

The societal importance of plastics contrasts with the carelessness with which they are disposed. Their superlative properties lead to economic and environmental efficiency, but the linearity of plastics puts the climate, human health, and global ecosystems at risk. Recycling is fundamental to transitioning this linear model into a more sustainable, circular economy. Among recycling technologies, chemical depolymerization offers a route to virgin quality recycled plastics, especially when valorizing complex waste streams poorly served by mechanical methods. However, chemical depolymerization exists in a complex and interlinked system of end-of-life fates, with the complementarity of each approach key to environmental, economic, and societal sustainability. This review explores the recent progress made into the depolymerization of five commercial polymers: poly(ethylene terephthalate), polycarbonates, polyamides, aliphatic polyesters, and polyurethanes. Attention is paid not only to the catalytic technologies used to enhance depolymerization efficiencies but also to the interrelationship with other recycling technologies and to the systemic constraints imposed by a global economy. Novel polymers, designed for chemical depolymerization, are also concisely reviewed in terms of their underlying chemistry and potential for integration with current plastic systems.

## 1 Introduction

Synthetic polymers are everywhere.^1,2^ Tuning monomer functionality, molecular weight, topology, and composition enables an unrivalled breadth of applications in textiles, electronics, healthcare, transportation, and packaging.^3^ The benefits in the resultant improvements on the human conditions, from the democratization of ownership to improvements in food safety and human health, are profound.^3−5^ Yet 91% of plastic ever made has been discarded or incinerated,^6^ leading to air quality crises from open burning,^7^ marine pollution impacting aquatic life,^8^ and pervasive microplastics.^9^ The overall contribution of plastic consumption to climate change is significant; by 2050, plastic production and incineration could require up to 13% of the remaining carbon budget needed to limit global warming to 1.5 °C.^10,11^

**Figure 1:**
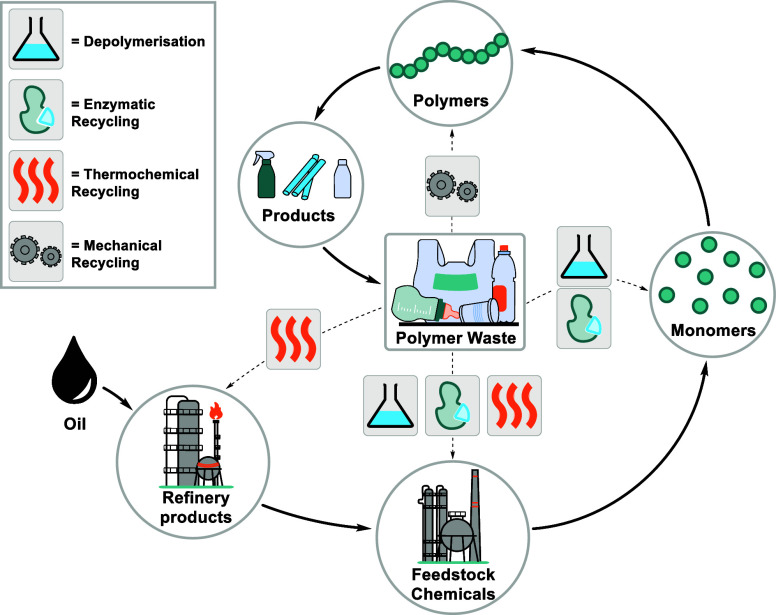
Different recycling technologies offer different entry points into a plastics circular economy, producing variable sized circular loops depending on feedstocks and efficiencies.

This dichotomy necessitates a plastics circular economy, equally motivated by improved environmental outcomes and recovery of significant economic value ($80–120 billion lost in plastic packaging in 2016).^12^ A future sustainable plastics system requires a combination of reduction, reuse, redesign, and recycling, enabled by consistent policies that retain benefits and understand social practice.^13^ Of these, recycling is the most complex and contentious, as if done sustainably could reduce waste and displace raw material consumption, yet if done poorly can precipitate unintended environmental consequences. Global postconsumer plastic recycling rates are stubbornly low (9% in 2019).^10^ Mechanical recycling (i.e., using heat and shear to melt plastic waste and reform it as new products) dominates,^14^ as it is cost-effective and can significantly reduce negative environmental impacts.^15−18^ However, it requires highly segregated feedstocks and does not work for all polymer types; other recycling approaches are necessary (Figure 1).

Chemical recycling, an umbrella of technologies for the deliberate conversion of polymer waste into small molecules, is growing in both academic and commercial interest.^19^ Chemical recycling embodies two main technological strategies. The first is thermochemical recycling, which primarily uses heat to break down polymers into small molecules. As a process, it is largely nonselective and produces a mix of monomers, oligomers, feedstock chemicals, and fuels. The second important chemical recycling strategy is chemical depolymerization, where polymers can be selectively converted into monomers or targeted chemicals, usually achieved by the action of solvents, catalysts, and heat. Commercial deployment of chemical recycling is limited: as of 2021, only 2.5 million tonnes of capacity was installed, less than 1% of annual global plastic production.^20,21^ Improving chemical recycling energy efficiency, economics, and selectivity are a focus for research, in the hopes of spurring wider commercial adoption.^22,23^

**Figure 2:**
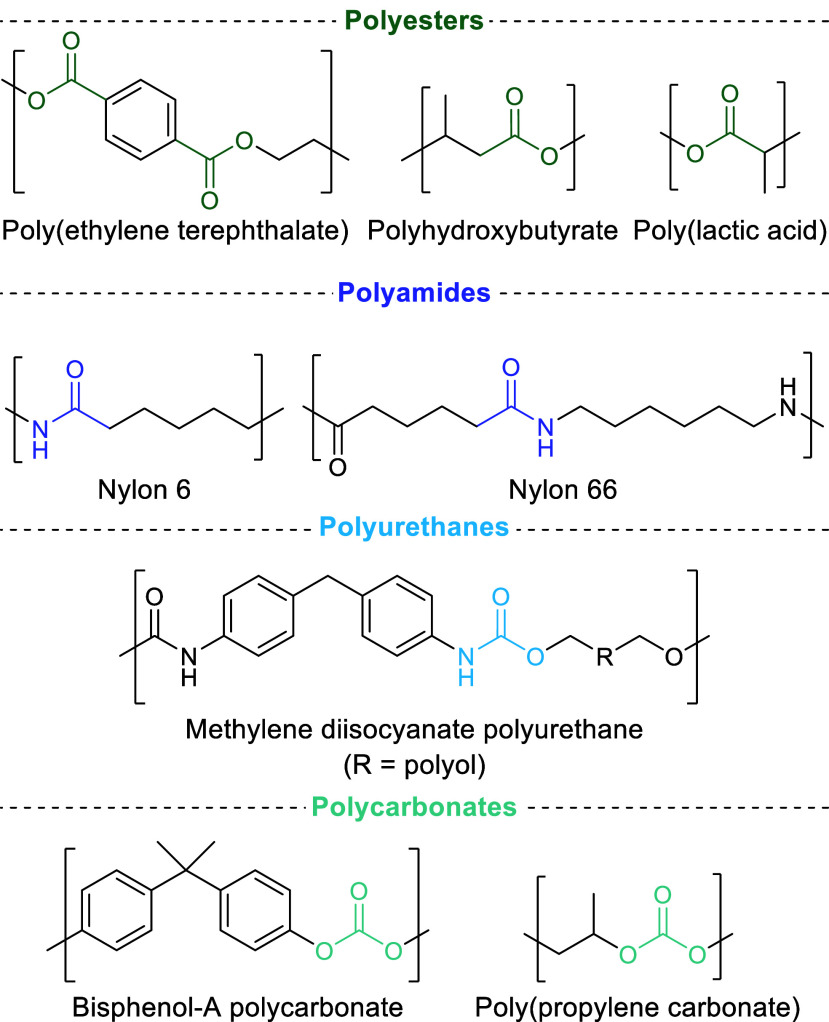
Four commercial polymer types discussed in this review. The functional groups typically attacked/cleaved during depolymerization are highlighted.

The chemical structure of a polymer is key to determining the efficiency of these different potential fates. Polymers with carbon–carbon backbones, such as polyethylene and other polyolefins, depolymerize at extremely high temperatures, and monomer selectivity is low due to random chain scission,^24^ with the strength of these bonds facilitating mechanical recycling. Polymer backbones containing C–X bonds (where X is a heteroatom) are more prone to selective cleavage and can facilitate depolymerization under milder conditions.^25^ Depolymerization research is therefore focused around polyesters, polyamides (PAs), polyurethanes (PUs), and polycarbonates (PCs) (Figure 2). Depolymerization research also interfaces with new polymer design, where monomer/polymer systems have been built with chemical recyclability in mind.^26^

Crucially, different end-of-life fates can be applicable with the same plastic waste feedstocks. Systemic approaches are needed to resolve these overlapping end-of-life fates and prioritize target streams for depolymerization strategies.^27^ This research gap is of interest, as proposed fates are often presented as a panacea for particular materials types. Optimally applied recycling technologies should work in concert to maintain polymers in the highest value condition with the lowest input energy. Applying a holistic approach to techniques like selective depolymerization, by considering how depolymerization will integrate with current and future waste systems,^28^ is also key to derisking commercial strategies. This includes pathways where the formation of a monomer feedstock would enable a genuinely circular economy as well as polymer “upcycling” strategies where an alternative product is formed.^29−31^ Upcycling is defined as the conversion of polymer waste to molecules of increased value. The definition of value is, at times, poorly defined or contentious. Evidence from techno-economic analysis (TEA) or life cycle analysis (LCA) can demonstrate the environmental or economic preferability for production from plastic waste over existing production routes.^32^ As the majority of proposed upcycling examples discussed herein lack a quantitative approach to value creation, quotation marks will be used to clarify “upcycling” has not been formally assessed.

The scope of this review is to critically consider the selective chemical depolymerization of commercial and novel polymers and explore how chemical recycling technologies can integrate into end-of-life systems. Harmony among recycling technologies is essential, and thus this review also features a brief overview of the strengths and limitations of mechanical, nonselective thermochemical, and enzymatic recycling to help identify where depolymerization could or should be applied. This review captures recent developments and covers publications from 2018 to 2023, though older resources are drawn upon where it proves informative.

### 1.1 Mechanical Recycling

The vast majority of plastic recycling is mechanical (over 99% in Europe).^33^ The process includes: collection of plastic waste, sorting, cleaning, granulation to flakes, and subsequent extrusion under high heat and shear, where polymer chains are remolded into a recycled plastic product. Significantly deeper technical elaboration can be found elsewhere.^14,34,35^ Environmental advantages of mechanical recycling vs incineration or landfill include significantly reduced raw material consumption and production-associated impacts (e.g., greenhouse gas (GHG) emission and water use). The technology is cost-effective and economically sustainable, although variability in oil prices and associated subsidies can exacerbate these risks.^36^ The efficiency of the process is tied to recyclate quality; better recyclate is obtained by improving sorting, washing, and extrusion.^37^

There are notable technical limitations of mechanical recycling. Thermoset polymers, which comprise 15–20% of global polymer production, cannot be effectively mechanically recycled.^38^ Mechanical recycling is sensitive to contaminants and mixed polymer types (e.g., multimaterials), necessitating complex and costly waste management systems.^39^ Even with clean, pure polymer waste, extrusion can cause polymer chain degradation as the high heat and shear induce polymer chain scission and branching depending on both polymer backbone and topology.^40−42^ Additives, used to regulate polymer properties, can both negatively and positively impact on this degradation and on end-of-life impacts, but is a neglected area of scientific inquiry.^34,43^ Mechanical recyclate is typically not suitable for more stringent applications (i.e., food-grade and medical-grade applications) as strict contaminant control is a common regulatory requirement.^44^

**Figure 3:**
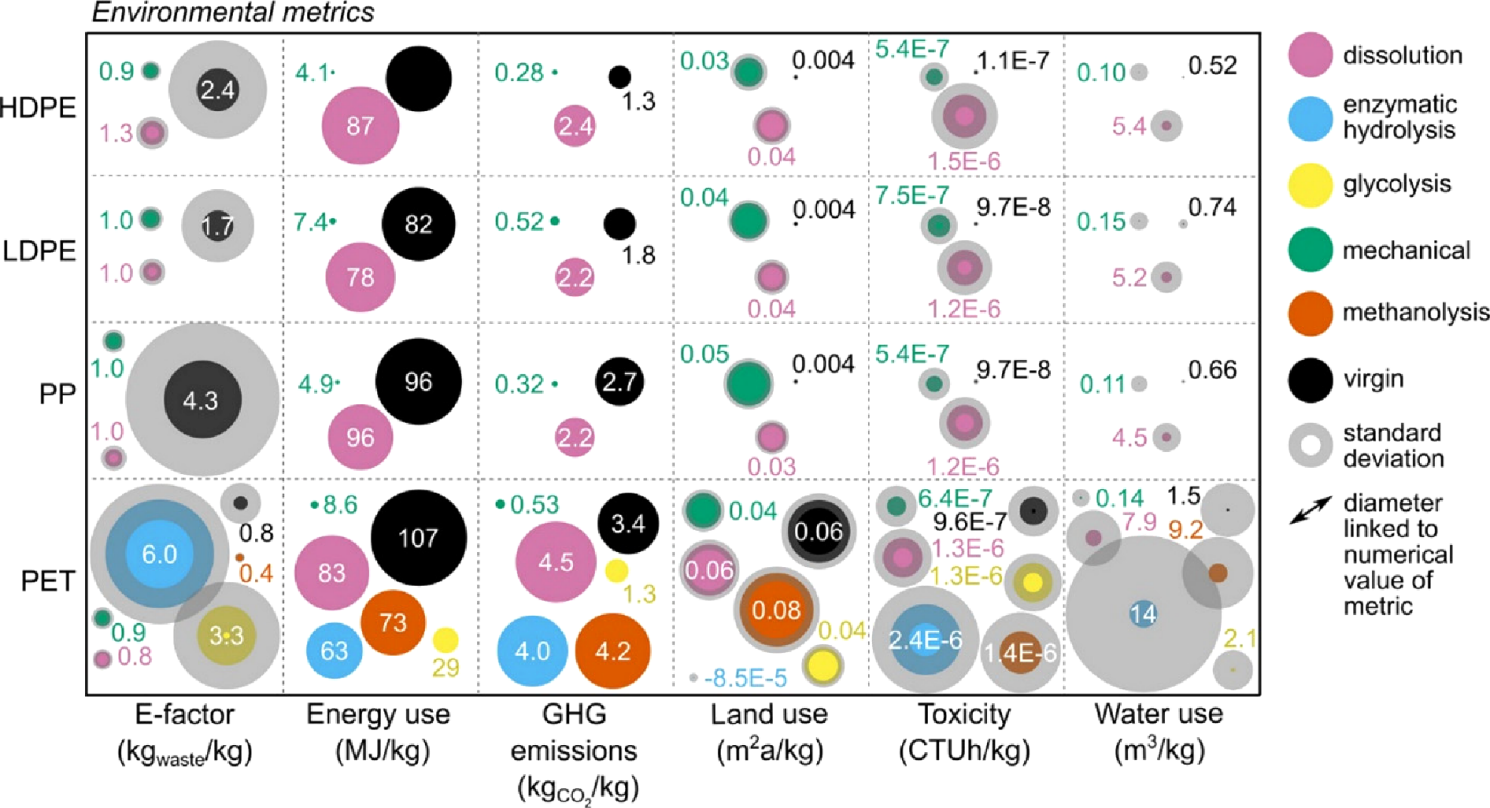
Comparison of the environmental impacts of recycling technologies for HDPE, LDPE, PP, and PET. Mechanical recycling is shown in green. Reproduced with permission from ref (47). Copyright 2023 American Chemical Society.

With mechanical recycling’s complex mix of strengths and weaknesses, analyses like LCA and TEA help evidence sustainable applications. Meys et al. found, for optimal global warming impacts, poly(ethylene terephthalate) (PET), high-density polyethylene (HDPE), low-density polyethylene (LDPE), polypropylene (PP), and polystyrene (PS) should be mechanically recycled; if incinerated, energy recovery in cement kilns is preferred.^45^ Other analyses corroborate that mechanical recycling leads to lower GHG emissions, energy and water usage than comparators, including depolymerization.^17,18,46^ Uekert et al. compared recycling technologies for HDPE, LDPE, PP, and PET and found mechanical recycling offered energy usage and GHG emissions 3–10 times lower than alternatives (Figure 3) (dissolution, glycolysis, methanolysis, enzymatic) and was more cost-effective.^47^

However, more complicated plastic waste streams may be more sustainably recycled by thermochemical or depolymerization approaches. Contaminated or mixed polymer wastes can be thermochemically recycled if processes are shown to be less sensitive to organic contaminants and mixed polymer types. This needs to be carefully designed, as thermochemical processes are expensive, energy intensive, and their products are not necessarily circular, releasing GHGs and losing material value. Depolymerization may be more complementary to mechanical recycling for heteroatom-containing polymer backbones. Additives, which preclude mechanical recyclate from food/medical applications, can be removed during depolymerization; direct monomer recovery is possible, allowing virgin-quality material to be prepared; mixed heteroatom-containing polymers may be sequentially depolymerized, with secondary sorting steps removed. As estimates suggest that the EU mechanical recycling rate would plateau at 49% by 2030, with global rates likely lower,^48^ a sustainable plastics future would require multiple technologies working in concert to create a circular system.

### 1.2 Nonselective Chemical Recycling

**Scheme 1:**
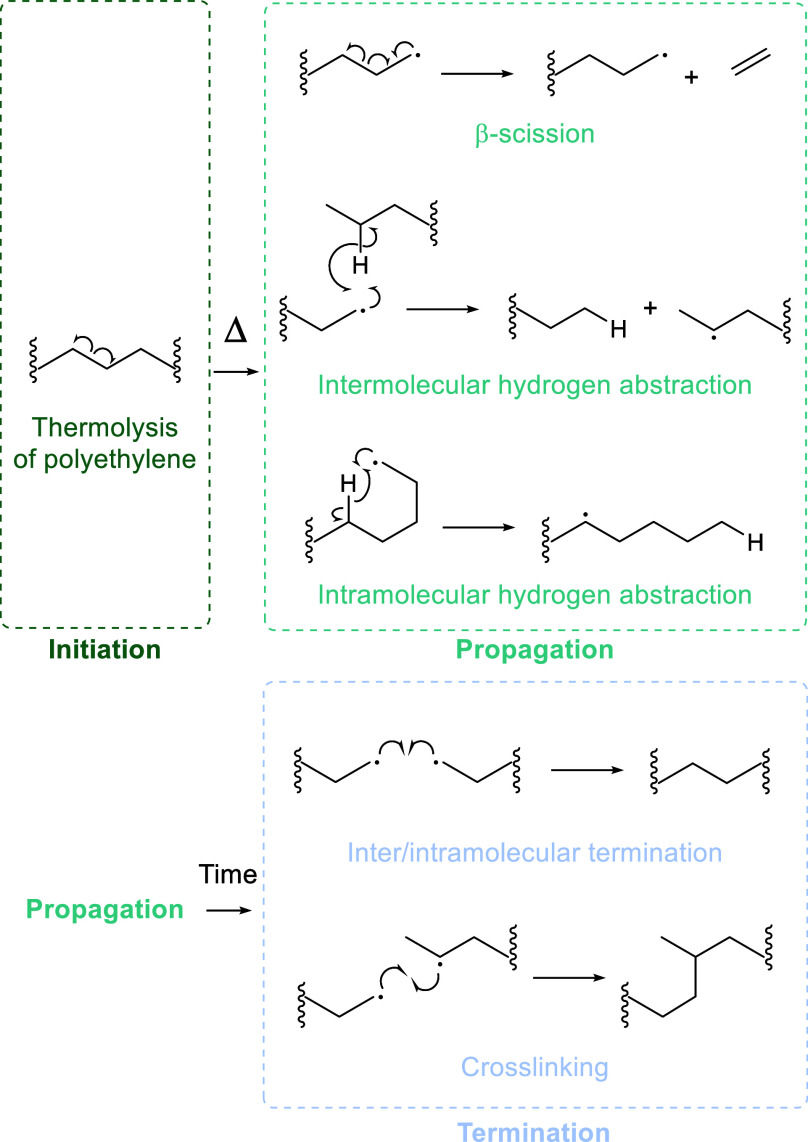
Initiation, Propagation, and Termination during the Thermal Degradation of Polyethylene

Nonselective chemical or thermochemical recycling technologies (pyrolysis, gasification etc.) use heat to convert plastic waste into chemical feedstocks. These strategies have been extensively reviewed.^14,49−54^ Thermochemical approaches typically utilize polyolefin waste, and polyolefins are difficult to selectively depolymerize due to a lack of a differentially cleavable bond (i.e., a chemical target). Polyolefins are polymers made from alkene monomers, like polyethylene or polystyrene. When significant heat is applied (400–700 °C) to polyolefins, their C–C and C–H bonds break in a statistical fashion via homolysis and a free-radical mediated initiation–depropagation–termination mechanism, similar to radical polymerization mechanisms (Scheme 1).^55^

**Scheme 2:**
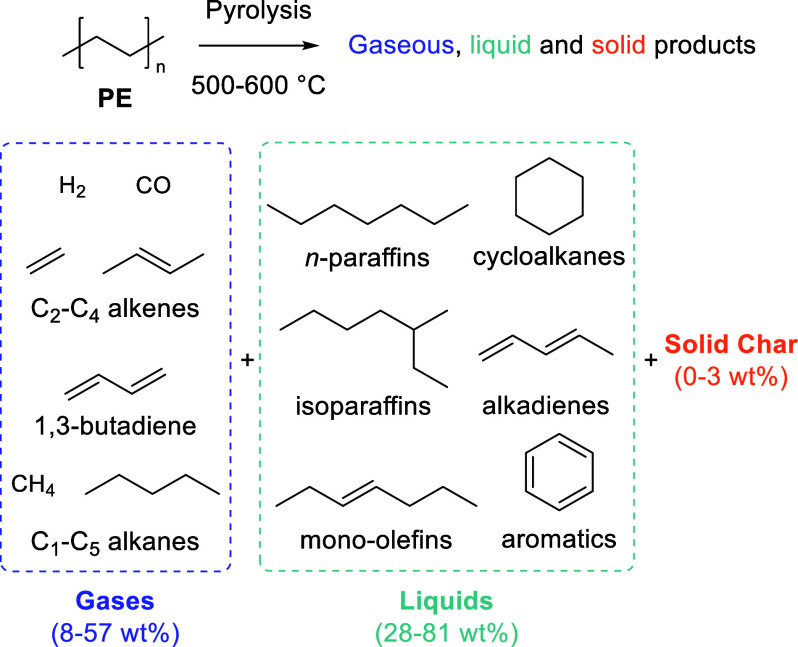
Polyethylene Pyrolysis Product Composition Phase and Weight Adapted with permission from ref (57). Copyright 2020 Wiley-VCH.

Thermochemical recycling efficiency is primarily controlled by feedstock quality and catalyst selection. Thermolysis of polyolefins produces a mixture of short, medium, and long-chain alkanes (C_1_–C_35_), alkenes, and aromatics (Scheme 2).^56,57^ For defined streams, selectivity improves. Polystyrene yields high proportions of styrene monomer and toluene,^58^ while thermolysis of nylon-6 produces the ε-caprolactam monomer in near-quantitative yields.^59^ A range of catalysts, typically heterogeneous, have been explored for thermochemical recycling. Through catalyst choice, the chemical composition and ratio of gas, oil, wax, and solid products collected can be controlled,^60^ at least at pilot scale. For example, Miandad et al. optimized polystyrene pyrolysis (450 °C, 76 min) to reach a 48.3% styrene yield.^61^

Thermochemical recycling has significant limitations, and its position in a circular economy is contentious. Although thermochemical approaches may be tolerant to organic contaminants (e.g., food residues), heteroatom/metal contamination in plastic feedstocks can alter the product composition of pyrolysis oil or damage plant equipment.^62^ The high energy use and costs of pyrolysis, hydrocracking, and gasification necessitate large-scale operation to be economical. Most distinctly, however, in most cases, thermochemical approaches do not yield monomers in high proportion, necessitating further chemical transformations or diversion to alternative products. In a genuinely circular plastics economy, from a mass balance perspective, polymer waste needs recycling to monomers, which is challenging to ensure. While thermochemical recycling may recover some value for mixed polyolefin waste, depolymerization creates pristine monomers.

### 1.3 Selective Chemical Recycling

Selective chemical recycling processes efficiently cleave chemical bonds to form targeted chemicals. Depolymerization, where the target is the parent monomers, is highly dependent on the chemical structure of a polymer backbone. Carbon main chains are challenging to depolymerize owing to a lack of polarity, and the low-lying highest-occupied molecular orbitals and high-energy lowest-unoccupied molecular orbitals of sp^3^-bonded carbon atoms. In 2017, of global polymer production, 61% by mass contained carbon backbones comprised of sp^3^-hybridized, carbon–carbon σ-bonds.^63^ For polyolefins, monomer reformation requires selectively reinstalling C=C bonds over competing scission reactions, meaning challenging cascades of catalytic reactions are required. Wang et al. reverted polyethylene to propylene by a combination of dehydrogenation, isomerization, and ethenolysis.^64^ Low temperature (100 °C) and high propylene selectivity (≥94%) were achieved, although catalyst deactivation occurred within 5 h. The thermodynamic difficulty of depolymerizing polyolefins remains significant and has not been assessed for its sustainability metrics.

Polymers which contain a chemical target, in the form of a cleavable functional group, are the most readily depolymerizable. Heteroatoms (i.e., N, O, S) in polymer main chains, introduce polarized C–X bonds between more electronegative atoms (O or N) and carbon. These C–X bonds are especially susceptible to nucleophilic attack.^24^ Chemical targets include esters, carbonates, amides, urethanes, and ethers, with each being integral to important class(es) of commercial polymers. This review will focus on published discoveries in the selective chemical recycling of these different polymer families.

## 2 Poly(ethylene terephthalate)

**Scheme 3:**
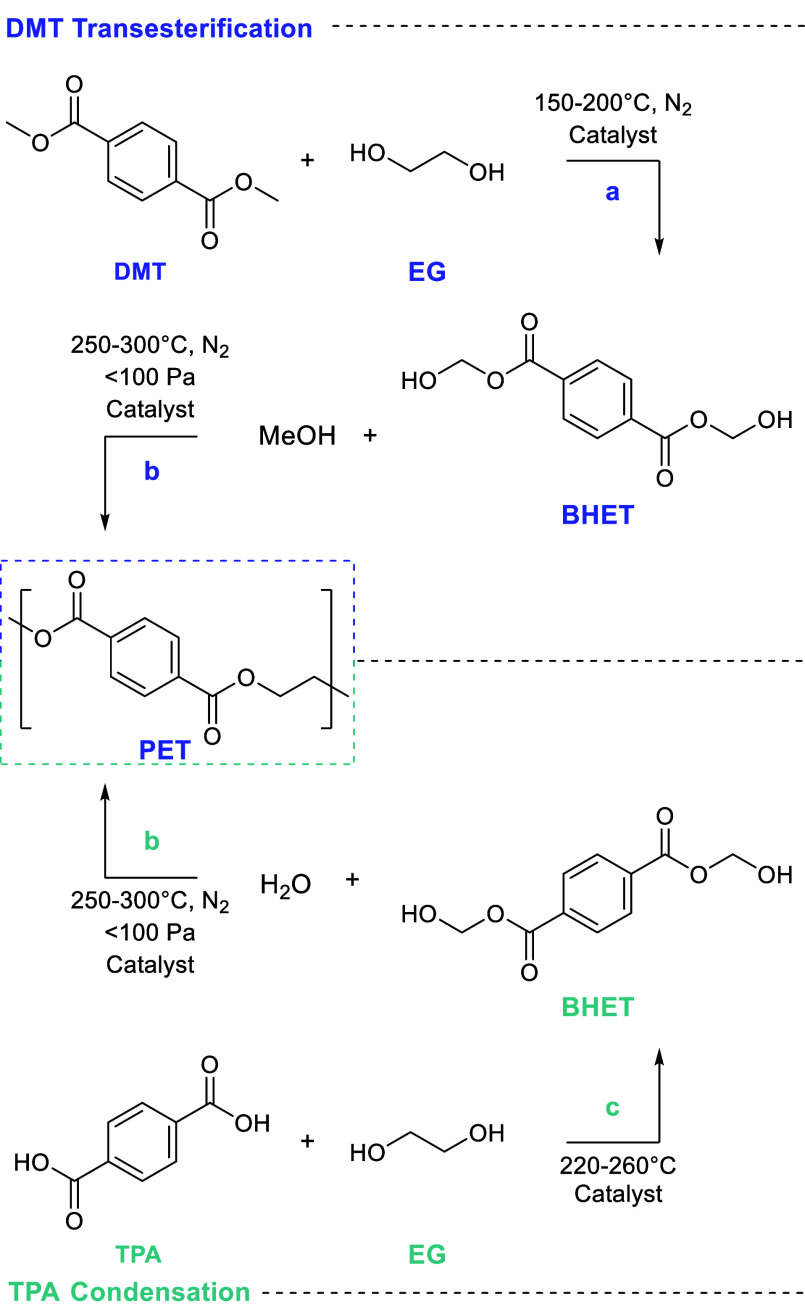
Production of PET by Two Main Commercial Methods (a) Catalytic transesterification and methanol formation by amine, metal alkoxide, and metal acetate catalysts. (b) Polycondensation using Sb, Ge, Ti, or Pb catalysts at high temperatures and reduced pressures. (c) Esterification of TPA and EG with a transesterification catalyst. Adapted with permission from ref (65). Copyright 2018 Wiley-VCH.

Poly(ethylene terephthalate) is the most widely used polyester globally, with an estimated 2021 consumption of over 65 million tonnes.^24^ The main applications of PET are in textile and packaging production. Commercially, PET has mainly been synthesized by two methods: dimethyl terephthalate (DMT) transesterification and terephthalic acid (TPA) polycondensation (Scheme 3).^65^ Multiple depolymerization strategies are available for PET, namely solvolyses in nucleophilic solvents (e.g., hydrolysis, glycolysis, aminolysis, ammonolysis, and methanolysis). Depending on the method, depolymerization of PET can produce: bis(2-hydroxyethyl) terephthalate (BHET) by glycolysis, dimethyl terephthalate by methanolysis, and terephthalic acid by hydrolysis. Each of the main depolymerization chemistries of PET is discussed herein.

### 2.1 Glycolysis

**Scheme 4:**
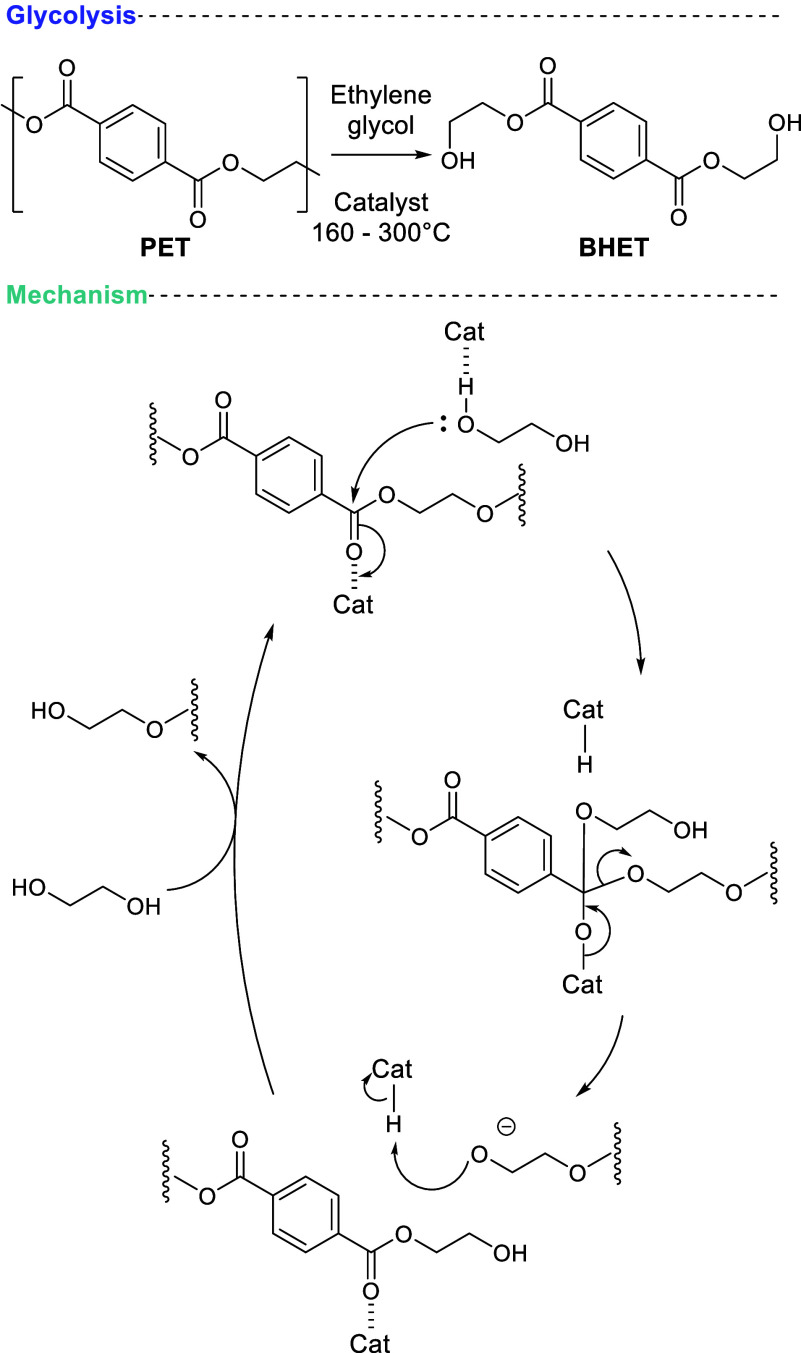
Typical Reaction Conditions for PET Glycolysis and an Example Catalytic Cycle Showing Dual Activation of a Glycolysis Catalyst Adapted with permission from ref (66). Copyright 2021 Royal Society of Chemistry.

Glycolysis is a transesterification reaction between PET and a glycol. Ethylene glycol (EG) is the most reported glycol and its use forms BHET, which is repolymerizable to PET by polycondensation (Scheme 3).^66^ Various glycolysis catalysts have been explored and often catalyze reaction via dual activation of the PET carbonyl and EG alcohol (Scheme 4).

An inherent challenge of glycolysis is the poor solubility of PET in ethylene glycol at room temperature. High glycolysis temperatures (160–300 °C) not only overcome the activation energy barrier but also enhance PET swelling in EG and encourage dissolution. Inclusion of cosolvents like dimethyl sulfoxide (DMSO), *N*-methyl-2-pyrrolidone, and nitrobenzene alongside EG can facilitate complete depolymerization in as little as 5 min.^67^ Addition of an anisole cosolvent also enabled lower glycolysis temperatures of 153 °C with similar BHET yield (>80%).^68^ Density functional theory (DFT) (a computational technique for probing reaction mechanisms) and ^1^H/^13^C NMR spectroscopic evidence suggested that anisole stabilized glycolysis transition state(s) and reduced activation energy requirements. Specifically, π–π interactions between anisole and PET and electron donation from the anisole methoxy group were vital. The solubility of PET in EG decreases with increasing PET molecular weight, making this a key parameter for comparison of glycolysis results.

The interplay between solubility and temperature means glycolysis processes have complex kinetics. For instance, one report on NaOEt-catalyzed glycolysis highlighted the temperature-dependent crossover between heterogeneous and homogeneous kinetic regimes.^69^ At 160–170 °C, glycolysis followed a shrinking-core kinetic model, whereas at 170–197 °C, a reversible first-order kinetic model was applicable. Shrinking-core kinetics have been reported elsewhere at system-specific temperatures.^70−72^ Through glycolysis processes, a mixture of both kinetic regimes often applies. With all else equal, the rate of homogeneous glycolysis is much greater than of heterogeneous due to dependence on volume rather than surface area. As such, PET particle size controls the time for homogeneity to be achieved, with rate inversely proportional to particle diameter.^72,73^

Glycolysis can be performed with or without a catalyst. In the absence of a catalyst, harsh conditions are required (≥300 °C and ≥1.1 MPa pressure).^74^ As such, catalytic approaches are more common. Catalysts used can be hetero- or homogeneous. Examples include metal acetates, oxides, and alkoxides as well as ionic liquids (ILs) and organic bases. Lewis acid zinc catalysts are effective at promoting glycolysis. In particular, zinc acetate, halides, and oxides have been reported.^60^ Zinc is also cheap and abundant; one report even sourced zinc oxide from spent car batteries.^75,76^ Across catalysts used, BHET yields of 70–90% are typical. Obtaining higher, near-quantitative yields of BHET is challenging as, in solution, the equilibrium between BHET and its dimer is very fast. Still, enhanced yields of >90% have been a focus of research, with hopes to boost the commercial viability of glycolysis. For example, Cao et al. achieved a 94.5% BHET yields using Mo/Co-doped ZnO nanosheets.^77^ Beneficially, the catalyst surface could be regenerated by heating to 500 °C to clear oligomeric PET, enabling catalyst recycling.

**Figure 4:**
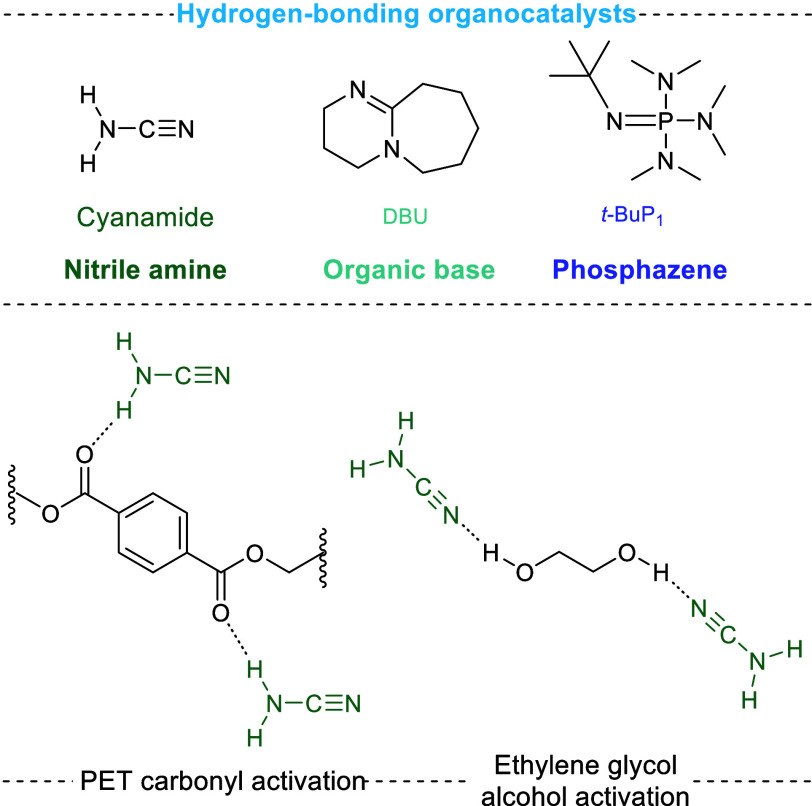
Dual activation by hydrogen bonding of PET by cyanamide. Phosphazenes and organic bases shown at the top catalyze glycolysis by a similar mechanism.

Organocatalysts have been explored as glycolysis catalysts, which, compared to their metal counterparts, are less air- and moisture-sensitive and do not deplete non-renewable metal resources.^78−80^ Wang et al. used cyanamide, a simple organocatalyst, to achieve outstanding BHET yields of >95% at 190 °C over 2.5 h.^81^^1^H NMR spectroscopy and DFT evidence supported a mechanism by which cyanamide enhanced both the electrophilicity of PET and the nucleophilicity of EG through hydrogen bonding (Figure 4). Similarly, Fan et al. obtained a >92% BHET yield using a phosphazene catalyst,^82^ although the PET scale was small at 0.5 g and the crystallinity of the PET was unreported. Crystallinity is an important parameter for comparison between glycolysis results due to its dampening effect on PET swelling and catalyst diffusion.

PET waste is well mechanically recycled, and so glycolysis would best be applied with differentiation from mechanical recycling in its capabilities. For example, polymer waste contains legacy additives which impact the mechanical recycling process and subsequent material quality. Such additives could be removed from glycolysis products (BHET) by crystallization. Methods like cationic exchange resins and zinc electrodeposition can be used to remove zinc catalysts from BHET products, up to 99% of the zinc present.^83,84^ Catalyst reclamation when using Zn or other metal catalysts is often neglected in glycolysis publications. Glycolysis could also be advantageous over mechanical recycling in the treatment of multimaterial waste. PET textiles are another significant waste stream which has been underexplored. One example from Yang et al. used a NH_4_CO_3_ catalyst for PET glycolysis of mixed fabric waste.^85^ In situ degradation of NH_4_CO_3_ into ammonia and CO_2_ allowed their system to depolymerize PET from multimaterial fabrics, which also contained cotton and left this cotton intact.

There has been greater interest in commercializing glycolysis than in other solvolysis processes. In Italy, Gr3n use microwaves to promote base-catalyzed glycolysis of PET and polyamides; in Japan, JEPLAN recycles both PET fibers and bottles by glycolysis; Canada-based Loop Industries use hydroxide-catalyzed alcoholysis and are aiming to commission their first large-scale site in France by 2027; and in the Netherlands, Ioniqa has a small plant for iron-catalyzed PET glycolysis.^86−89^

Pairing the best performing glycolysis systems with rigorous analyses like LCA and TEA would be invaluable for identifying optimal systems and environmental hotspots requiring improvement. Luo et al. used TEA and LCA to analyze the potential benefits of microwave heating to glycolysis.^90^ Using microwaves was shown to be economically and environmentally advantageous compared to traditional heating methods during glycolysis. Overall, examples of LCAs are scant in the literature compared to the number of glycolysis processes reported.

### 2.2 Hydrolysis

**Scheme 5:**
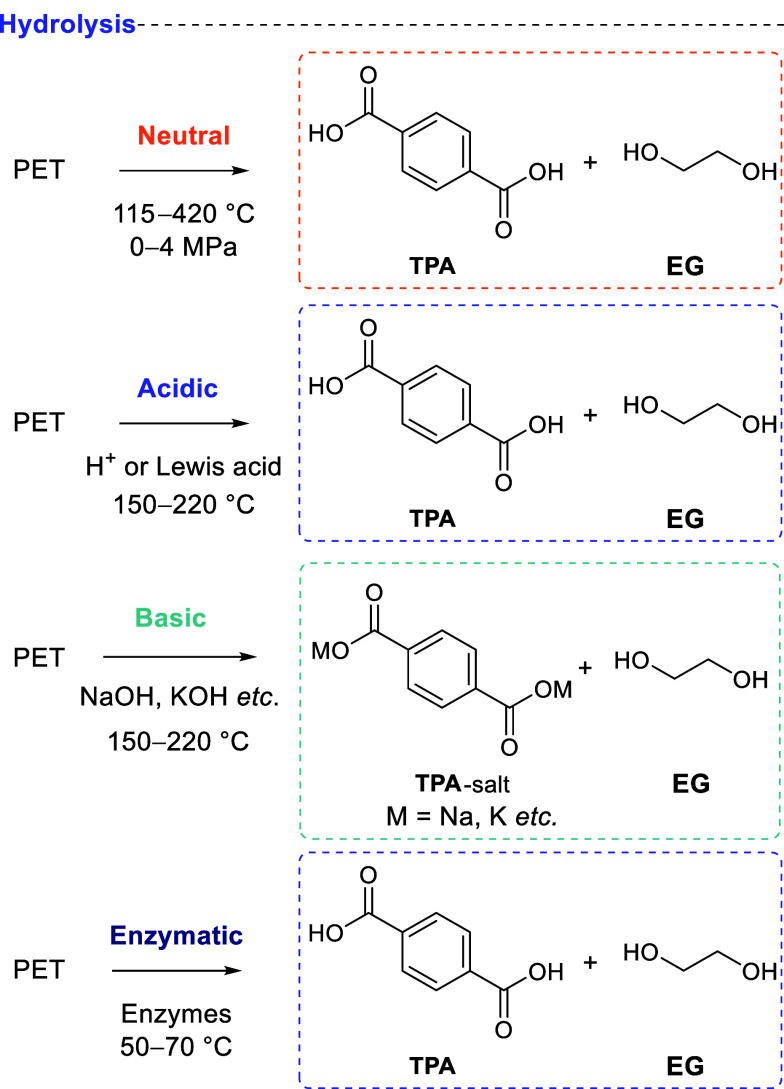
Four Hydrolysis Approaches, Differentiated by Catalyst Type and Conditions Used Adapted with permission from ref (92). Copyright 2003 Springer.

Hydrolysis is the cleavage of PET by water, yielding terephthalic acid and ethylene glycol. Hydrolysis is the primary environmental degradation pathway of PET released into wet environments, taking hundreds of days to even partially degrade PET.^91^ Purposeful hydrolysis can be done in the absence of a catalyst, neutral hydrolysis, with forcing conditions like high temperature (up to 420 °C) and/or pressure needed as water is a weak nucleophile. Most catalytic hydrolyses use acids, bases, or enzymes (Scheme 5).^92^ Each of these methods is discussed herein.

#### 2.2.1 Neutral Hydrolysis

Neutral hydrolysis reactions are done at neutral pH, often without a catalyst, at high temperature and/or pressure to achieve depolymerization. Pereira et al. investigated the effect of temperature, pressure, and water phase (e.g., saturated, supercritical) on the neutral hydrolysis of PET.^93^ By rapidly heating (5–10 °C s^–1^) their reactor toward 500 °C, 78% TPA yield was obtained in 1 min. The process had an impressively low environmental energy metric compared with similar works. By removing catalysts, neutral hydrolyses benefit from reducing the amount of product purification and waste generation.^94^

Challenges for neutral hydrolyses include the poor nucleophilicity of water and the low water-solubility of PET. Ezeanu et al. investigated the use of seawater as a hydrolysis catalyst.^95^ Naturally high in metal salts (Na, Mg, Ca etc.), TPA yields of up to 96% were attained using Atlantic Ocean water at 100:0.35 water to PET mass ratio. The metals present acted act as Lewis acid catalysts for hydrolysis (via carbonyl activation). The sustainability of neutral hydrolysis could most be improved by reducing water use and energy inputs.^96^ For example, Circ, based in Virginia (USA), has a process which uses subcritical water at a relatively mild 105–190 °C and 2.7–20.0 bar to recover TPA and cellulose from PET/cotton textile waste.^97^

#### 2.2.2 Acidic Hydrolysis

Acidic hydrolysis uses protons as the primary catalyst for hydrolysis. Protonation of the PET carbonyl activates it for nucleophilic attack by water. The adduct formed by nucleophilic attack then fragments, and these steps are repeated until TPA and EG are yielded.^98^ Due to PET’s hydrophobicity, the reaction is usually heterogeneous with shrinking-core model kinetics reported. Older examples used strong acids like sulfuric, nitric, or phosphoric, but their usage can be costly and generates stoichiometric waste.^99,100^ Instead, Lewis-acidic zinc chloride-catalyzed depolymerization has been reported to achieve >98% TPA yield after 8 h at 180 °C, with no stoichiometric acidic waste.^76^ However, testing to show no zinc had leached into the TPA was missing; zinc presence in TPA or BHET products is a common problem in PET hydrolysis and glycolysis.^83^ TPA itself has been used as an acid catalyst for hydrolysis.^101^ A TPA yield of 95.5% was obtained under optimized conditions. A high activation energy of 220 kJ/mol^–1^ was calculated, likely as TPA is a weak acid.

**Figure 5:**
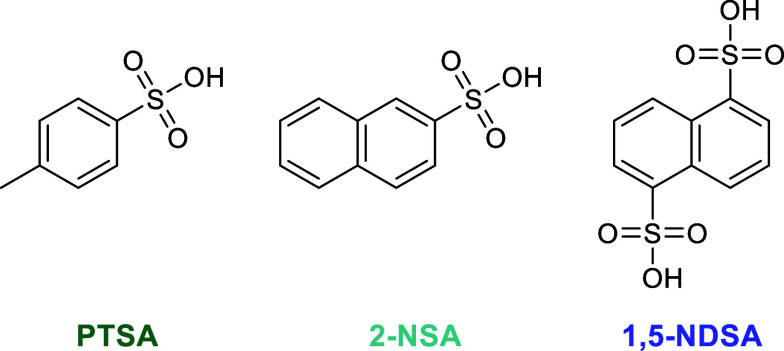
Structure of different aryl sulfonic acid catalysts.

Sulfonic acid catalysts have been investigated for the hydrolysis of PET. Yang et al. used *p*-toluenesulfonic acid (PTSA) and achieved a 96.2% TPA yield after 90 min at 150 °C, while maintaining this performance across 5 cycles.^102^ An in-depth probing into aryl sulfonic acid catalysts by Abedsoltan et al. was revealing.^103^ Using PTSA, 2-naphthalenesulfonic acid (2-NSA), and 1,5-naphthalenedisulfonic acid (1,5-NDSA) (Figure 5), >90% TPA yields were observed at 150 °C. However, longer reaction times were needed of 3, 3 and 8 h for PTSA, 2-NSA, and 1,5-NDSA respectively. Interestingly the bifunctional 1,5-NDSA was a slower and less active catalyst than PTSA. This decreased speed and activity was ascribed to a difference in affinity of the catalyst for the PET surface.

There is a dearth of LCAs covering any type of acidic hydrolysis. It is therefore hard to compare it with other depolymerization and recycling approaches in terms of sustainability. Against this trend, one report of acetolysis (cleavage by acetic acid) for PET waste demonstrated the process with PET textile waste.^104^ The acetolysis and subsequent purification process was able to remove color from the final TPA product and worked with a PET/nylon blend, a representative real-world waste textile case. The authors performed an LCA which suggested the process had comparable global warming potential (1.04 vs 0.96 kg CO_2_ equiv/kg PET chips) and non-renewable energy use (13 vs 13 MJ/kg PET chips) impacts to mechanical recycling in the EU, and performed much better than other PET depolymerization methods (glycolysis, methanolysis, hydrolysis).

#### 2.2.3 Basic Hydrolysis

Basic hydrolysis uses a strong base (e.g., OH^–^) to perform nucleophilic attack on the PET carbonyl. The initial product is a terephthalate/metal salt, which requires acidic workup to yield the desired TPA monomer. The additional workup and stoichiometric waste are drawbacks of basic hydrolysis. Efforts to design catalysts that lower the amount of base (NaOH, KOH) are needed.^105^ A further challenge faced is poor water solubility of PET. Ügdüler et al. overcame this solubility issue by including an ethanol cosolvent.^96^ Under optimized conditions (60:40 EtOH:H_2_O, 5 wt % NaOH and 80 °C), a 95% TPA yield was observed after only 20 min. The system was tested on postconsumer PET waste of differing types and a decreased product yield of 60% was observed. This difference between pure and postconsumer waste highlights the importance of designing robust systems able to meet the demands of industrial application.

Academic examples of basic hydrolyses are scant. Arias and Thielemans reported on a KOH-in-methanol system which used microwave heating to depolymerize PET samples at 120 °C, in as little as 1 min.^106^ Microwave heating was suggested to give short reaction times by exponentially increasing the reaction pressure and heating the solution uniformly. However, this short reaction time may partially be an artifact of scale; cases at >10 g showed longer reaction times.^107^ Other work has looked at ball-milling as a solvent-free method for basic hydrolysis. Solvents including water are often a significant environmental outlay in industrial processes, so from this point of view, the benefits are clear.^108,109^ Štrukil reported on 93% TPA yields after ball-milling for 1 h with 1 equiv of NaOH to 0.5 g of PET.^110^

Efforts to commercialize basic hydrolyses include Switzerland-based DePoly, who use basic/metal oxide cocatalysts along with UV light to depolymerize PET with reportedly high tolerance for contamination.^111^

#### 2.2.4 Enzymatic Hydrolysis

**Scheme 6:**
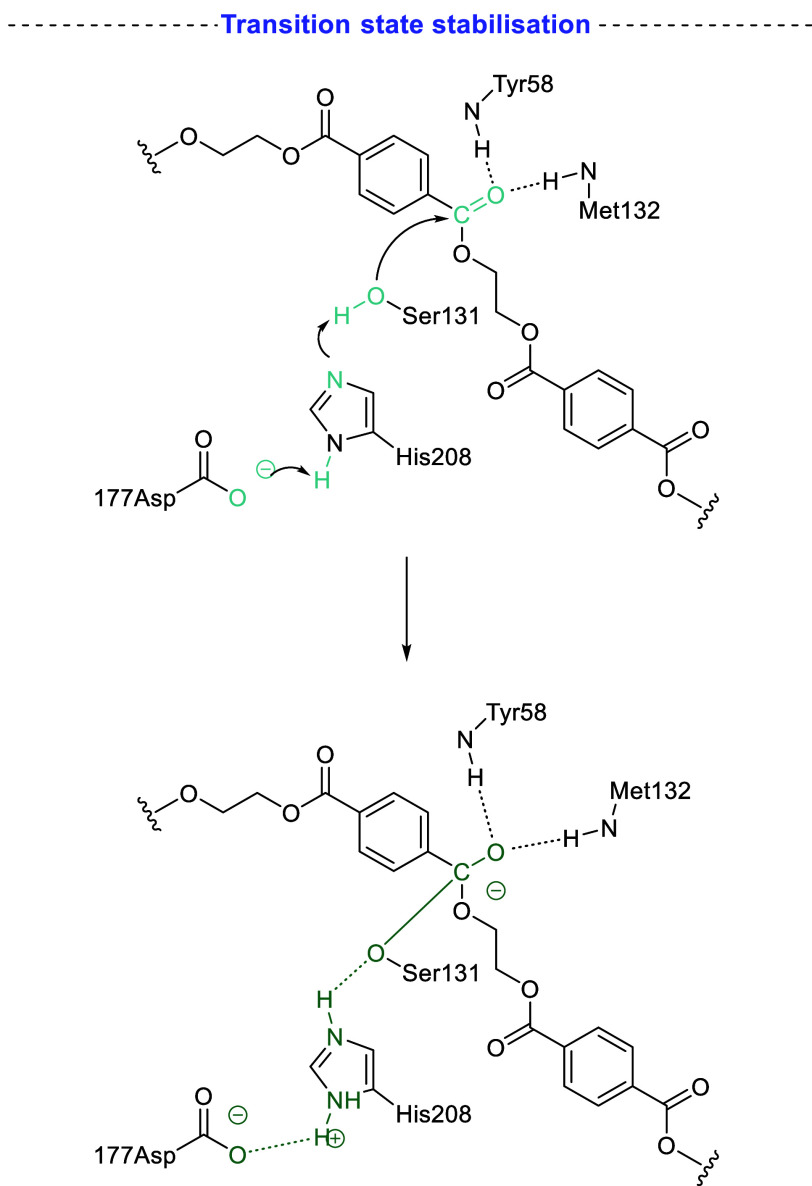
DFT-Simulated Action of Is-PETase during Hydrolysis of PET Adapted with permission from ref (118). Copyright 2021 American Chemical Society.

Enzymatic hydrolysis is a promising method to selectively produce monomers under mild conditions while avoiding chemical waste (e.g., organic solvents). For more detailed discussion, see the extensive reviews on the topic.^112−115^ Enzymatic hydrolysis of PET was pushed to the fore by the 2016 discovery of a bacteria (*Ideonella Sakaiensis*) which produced a PET-degrading enzyme known as Is-PETase.^116^ Mechanistically, the action of the Is-PETase enzyme was proposed to be similar to chemical depolymerization; nucleophilic attack of a serine-linked O^–^ anion on the carbonyl carbon.^117^ Work by Jerves et al. elaborated on this mechanism by probing with DFT (Scheme 6).^118^ Several Asp residues were identified in the active site for mutation that could improve activity.

Development of more active and selective enzymes for depolymerization is a focus of research. For example, a leaf-branch compost cutinase was identified with 10,000 times greater specific activity than Is-PETase.^119^ Tarazona et al. evaluated the activity of an Is-PETase triple mutant with increased thermal stability (*T*_m_ of 56.6 °C) on amorphous PET films.^120^ Temperatures above the glass transition temperature (*T*_g_) of PET gave greater surface porosity and improved catalytic rates and PET conversions (70% after 1 h at 50 °C). Additionally, polymer thickness and crystallinity were both inversely proportional to the rate of enzymatic degradation.

Enzymes are currently slow catalysts; reactions can take days to reach high yields, depending on the enzyme.^121^ This sluggishness is in part due to the size of enzymes and heterogeneous nature of their reactions. Adsorption efficiency is key to reaction rate as well as catalytic activity.^122^ Slower rate is also resultant of the lower temperatures used (enzymes typically denature at higher temperatures). Increasing the thermostability of enzymes can enable their use at more kinetically beneficial temperatures.^121,123,124^ Operating at temperatures above the polymer’s *T*_g_ is desirable as this allows better penetration of the enzyme within the polymer chains. Another recently discovered enzyme with PET-degrading properties, PHL7, has been shown to benefit from increased temperature stability by 17 single mutations.^125^

Much of the world’s PET waste is semicrystalline, and most enzymes negligibly degrade crystalline PET regions. Pretreatment steps can be incorporated to amorphize PET, although these steps come at the price of increasing complexity, cost, and energy demand. Kaabel et al. managed to obtain 50% TPA yield from a commercial, 36% crystalline PET sample.^126^ Key to their approach was spiking in fresh enzyme solution at regular intervals, suggesting enzyme deactivation was occurring. Once active and stable enzymes are found, more screening on real polymer waste needs performing to fill current knowledge gaps.^127^ Additives and contaminants present in polymer waste may hinder enzyme activity. For example, many flame retardants and UV stabilizers rely on metals which can bind and deactivate enzymes. The interplay between additives and enzymatic degradation is poorly understood.

With industrial-scale enzymatic hydrolysis on the horizon (a first commercial plant is expected in France by 2025),^128^ there is an acute need for evidence of its environmental or economic preferability. A combined techno-economic life cycle and socio-economic assessment by Singh et al. was illustrative.^129^ By evaluating a plausible case for an enzymatic recycling plant in the U.S., key drivers of environmental impact and economic cost were outlined. On the environmental side, electricity use was the main outlay driven by pretreatment and mechanical reprocessing steps. These could be allayed by finding enzymes that can depolymerize more contaminated, crystalline substrates.

The role enzymatic hydrolysis will play in a circular plastics recycling system is currently undefined. An ideal feedstock is pure PET waste, potentially diverting it from lower footprint mechanical recycling. Comparative LCAs indicate mechanical recycling has significantly lower energy use and environmental impacts than enzymatic hydrolysis.^47,130^ The power of enzymatic hydrolysis may indeed be rooted in selectivity, particularly to recover PET monomers from mixed waste streams. Looking to the future, Uekert et al. pointed out key research targets: increased process yields, lower pretreatment requirements, enzymes that tolerate high substrate-loading, and crystallinity.^131^ With such improvements, enzymatic hydrolysis could become an important technology for recycling PET, polycarbonates, and other polyesters.

### 2.3 Aminolysis and Ammonolysis

**Scheme 7:**
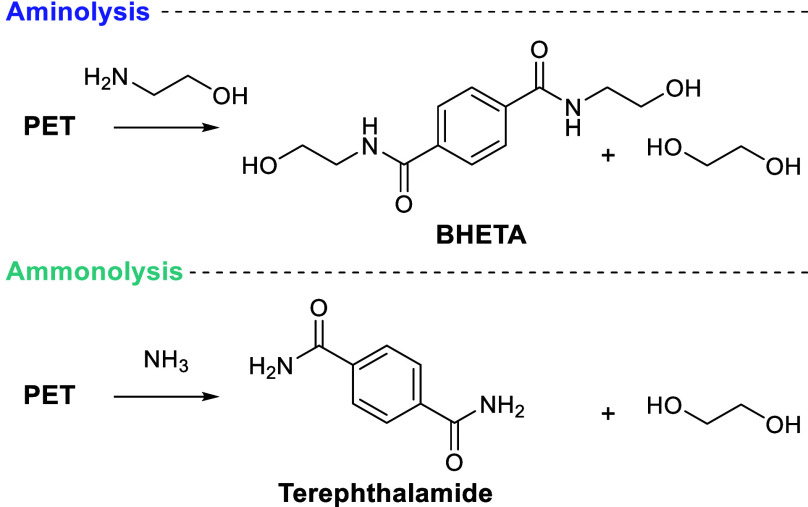
Aminolysis of PET with Ethanolamine to Form BHETA and Ethylene Glycol and Ammonolysis to Yield Terephthalamide and Ethylene Glycol

Aminolysis and ammonolysis are transamination strategies, making use of an amine and ammonia respectively, as nucleophile to degrade PET (Scheme 7). Ethanolamine is commonly used, which forms bis(2-hydroxy ethyl)terephthalamide (BHETA) and ethylene glycol as the primary products. Researchers seek to exploit the increased nucleophilicity of nitrogen compared to oxygen to afford rapid, mild reactions. Early studies identified a range of suitable catalysts, both homogeneous: acetic acid,^132^ potassium sulfate,^132^ 1,5,7-triazabicyclo[4.4.0]dec-5-ene (TBD),^133^ zinc acetate,^134^ and heterogeneous.^135^ Recent publications however are few, mirroring a lack of commercial examples of either reaction with PET waste.^136^

The strength of the amine nucleophiles allows both catalytic and noncatalytic systems to have short reaction times. Demarteau et al. found 93% BHETA yields in just 15 min using a TBD:methanesulfonic acid (MSA) catalyst.^137^ These yields were modestly (5%) better than those obtained with no catalyst. The degradation products were subsequently polymerized into poly(ester amides) with reasonable molecular weight (Number-average molecular weight *(M*_n_) = 10 kDa) and control (*Đ* = 1.72). Applications for poly(ester amides) are not well-defined, so the feasibility of this “upcycling” approach for commercial-scale recycling of PET remains unclear.

As repolymerization of aminolysis products into PET is impossible, numerous examples “upcycle” the products. Diverse applications including electromagnetic interference foams, epoxy hardeners, hydrogel adsorbents, and Schiff bases have been explored.^138−141^ Hedrick and colleagues used a catalyst-free aminolysis of PET at 120 °C over ∼2 h to produce antimicrobial polyionenes.^142^ The system appeared robust as postconsumer PET waste was used, however scale was not indicated. Microwave-heating-assisted aminolysis has been investigated and shown to be a catalyst-free and energy-efficient system.^143^ Heterogenous aminolysis catalysts have also been reported. Vinitha et al. made use of ZnO nanoparticles and Gopal et al. of clay-supported phospho-acids.^144,145^ In both cases, good BHETA yields were obtained and the catalysts were recyclable. Ammonia can be even more reactive than amines, although there are few reports. Xu et al. produced terephthalonitrile by pyrolyzing PET in an ammonia atmosphere^146^ at high temperatures (500 °C) and with low selectivity (terephthalonitrile yield <50%).

### 2.4 Methanolysis

**Scheme 8:**
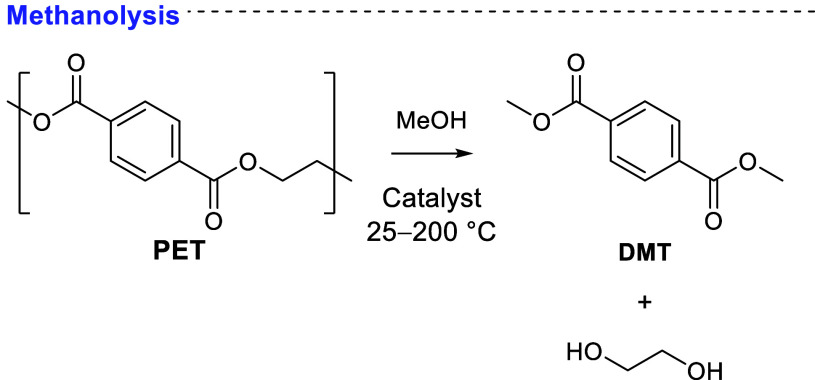
Typical Methanolysis Typical Conditions Catalysts include bases, Lewis acids, and ionic liquids.

Methanolysis is a transesterification between methanol and PET (Scheme 8). The products are dimethyl terephthalate and ethylene glycol. Similarly to glycolysis, PET is poorly soluble in methanol at lower temperatures.^147^ Supercritical methanolysis has been reported in academic literature and industrially applied.^148−150^ At lower temperatures (<150 °C), the reaction remains heterogeneous, with PET particle size a critical parameter. One study found a particle size of 127.5 μm to be a cutoff point for optimum performance.^151^ Size-reduction treatments of PET waste add to the economic and environmental burden of prospective methanolysis processes. Pretreatment of PET by extrusion and melt-quenching to lower crystallinity has also been reported to improve DMT yields by 8–19%.^152^

PET solubility in methanol can be improved by inclusion of a cosolvent. For example, 20 vol % of toluene cosolvent in an aluminum-catalyzed methanolysis process led to a 24% greater DMT yield.^153^ Use of chloroform as cosolvent, in concert with microwave heating, has also yielded short reaction times.^154^ After just 60 min, Hofmann et al. reported 98% DMT yields at 140 °C. A scaled-up (10.5 g PET) version of the reaction gave 69% isolated yield after 2 h. Scale influences rate by changing the specific surface area on which a catalyst can act. Tang et al. informatively probed the mechanism by which cosolvents increase reactivity.^155^ DFT calculations suggested, using BHET instead of PET, that polarity matching between solvent, cosolvent, and substrate significantly enhanced diffusion of methanol the substrate surface. A similar phenomenon was observed by Liu et al. in supercritical methanolysis, with CO_2_ enhancing the solubility of MeOH in PET.^156^

**Figure 6:**
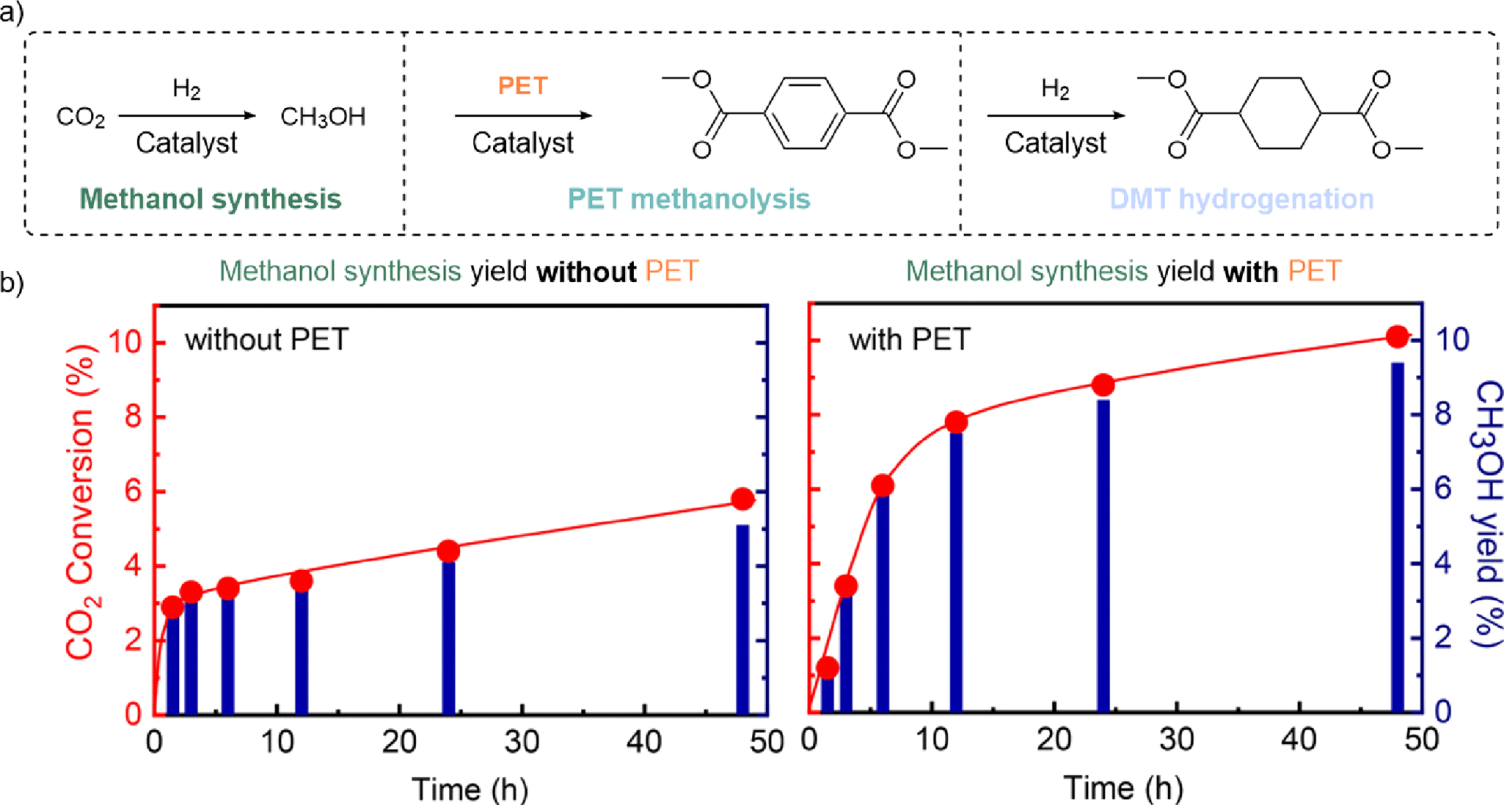
(a) One-pot CO_2_-to-DMT reaction broken down into its constituent reactions: methanol synthesis, PET methanolysis, and DMT hydrogenation. (b) Conversion plots that demonstrate the driving impact that PET consumption has on methanol yield and CO_2_ consumption. (a) Adapted and (b) reproduced with permission from ref (157). Copyright 2021 John Wiley & Sons.

Methanolysis depolymerizations operate under equilibrium. Following Le Chatelier’s principle, the position of equilibrium can be constantly pushed to the right by consuming products (DMT, EG) as they form in one-pot processes. Li et al. demonstrated a transformation that utilized PET, CO_2_, and H_2_ to produce a dicarboxylated cyclohexane derivative (Figure 6).^157^ Tanaka et al. instead captured EG with dimethyl carbonate, which led to good yields of DMT (72–91%) and ethylene carbonate (69–86%) at 65 °C.^158^ Mild methanolysis conditions have been reported with respectable yields of DMT (71–86%) attainable at 70 °C using MeOK, K_2_CO_3_, or TBD as catalyst.^159^ In turn, the reaction time was 20 h. Pham and Cho obtained 93.1% DMT yields at 25 °C after 24 h, by including DCM cosolvent.^160^ Dependence on halogenated solvents like DCM is not environmentally preferable.

Heterogeneous catalysts have been applied to methanolysis processes. For example, bamboo leaf-ash was heat processed (via calcination) and used as a catalyst, as it contained reactive species (Mg^2+^, Mn^2+^, and O^2–^).^161^ The calcination pretreatment was energy intensive; 700 °C for 4 h. A common feature of heterogeneous systems is high operating temperature (200 °C), likely required to ensure PET dissolution and binding to the catalyst surface. Magnesium oxide and magnesium–pectin catalysts have been reported to give >90% DMT yields along with catalyst recyclability.^162,163^ A multistep but mild preparation of ZnO nanoparticles was reported by Du et al., with use of the nanoparticles demonstrating excellent DMT yields (>95%) after just 15 min, even at 10 g PET loading.^164^

A combined LCA and material flow analysis compared the impacts of virgin-PET production, methanolysis, hydrolysis, and mechanical recycling.^130^ Although mechanical recycling had the lowest energy use and GHG emissions, methanolysis was lower in water use in a fiber-to-bottle scenario. The majority of GHG emissions were incurred during the waste-to-monomer stage, suggesting optimization of the underlying chemistry of these processes (e.g., greater yields, lower temperature, more-active catalysts, effective and simple separation steps) can afford better sustainability outcomes. Furthermore, a main environmental driver was the use of natural gas to produce the methanol for methanolysis. Reducing the amount of methanol used would significantly lower GHG emissions. The chemical company Eastman are developing a 100,000-ton-per-year methanolysis plant for waste-PET treatment in Tennessee and are also planning to create a second 200,000-ton site in France.^165^

### 2.5 Ionic Liquids and Deep Eutectic Solvents

The term ionic liquids (ILs) refers to liquids composed of ions at or below 100 °C (at atmospheric pressure).^166^ ILs can serve as both solvent and catalyst in PET depolymerization. As catalysts, IL-anions hydrogen bond to PET and EG, increasing nucleophilicity by lengthening and weakening the O–H bond, and cations can activate the PET carbonyl.^167,168^ Molecular dynamics simulations suggest that by electrostatic and hydrogen bonding interactions, ILs increase PET swelling at higher temperatures, increasing EG access to the polymer chains in solution.^169^

**Figure 7:**
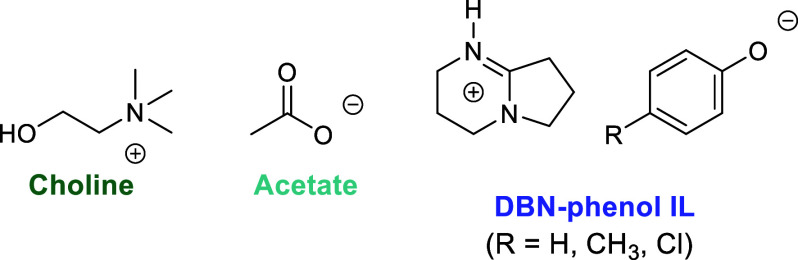
Structure of a choline acetate and DBN-phenol ionic liquid.

ILs have been applied across PET depolymerization approaches (glycolysis, hydrolysis, methanolysis).^170,171^ For example, Liu et al. demonstrated a series of choline-based ILs (Figure 7) suitable for glycolysis, reporting 85% BHET yields after 4 h at 180 °C.^70^ A relatively large catalyst loading of 5 wt % was needed. This need for high catalyst-loading has been documented elsewhere.^172−174^ Other systems include 1,5-diazabicyclo[4.3.0]non-5-ene (DBN) phenols (Figure 7), which have been reported as reusable up to 8 times,^71^ although the DBN-phenol was not separated from the EG between each run. ILs have also been incorporated into heterogeneous catalysts by grafting onto a graphene support.^175^

Deep eutectic solvents (DESs) are a class of ionic liquids with many similar properties. Formally, DESs contain a eutectic mixture of Lewis or Brønsted acid and base hydrogen-bond donors and acceptors.^176^ A eutectic mixture is the composition of two or more components which has the lowest melting point. Further review of DESs can be found elsewhere.^177,178^ For PET depolymerization, catalysis is enabled by a similar mechanism to ILs.

Typical performance of DES-catalyzed glycolysis or hydrolysis is not dissimilar to those discussed in previous sections. Liu et al.’s 1,3-dimethylurea/Zn(OAc)_2_ glycolysis catalyst gave 82% BHET yield at 5 g PET scale after 20 min at 190 °C.^179^ PET pellets were ground down to 40/60 mesh size (250–425 μm diameter), increasing PET surface area and reaction rate. This pretreatment grinding has been reported elsewhere with a similar resultant reaction duration.^180^ Moreover, in the case of Liu et al., inductively coupled plasma results after 5 recycles showed the zinc content had dropped to 25% of its original value, suggesting the zinc may be leaching into the product. Instances of “upcycling” were reported by further transformation of the products of DES-catalyzed depolymerization. Zhou et al. prepared dioctyl terephthalate, an alternative to the commercial plasticizer dioctyl phthalate, by alcoholysis in 2-ethyl-1-hexanol.^181^ Similarly Lee et al. screened a range of potassium-based DESs for the depolymerization of PET and one-pot synthesis of polyisocyanurate foams which are used as flame-retardant insulation.^182^

DESs have also been applied to catalyze PET hydrolysis and aminolysis.^183,184^ Other efforts have used DESs on polyester/cotton fibers.^185^ Wang et al. recovered microcrystalline cellulose from polyester–cotton blended fabrics using a choline chloride and PTSA-based DES.^186^ Li et al. used DBU-based DESs to catalyze glycolysis of PET from bottles.^187^ An 83% BHET yield was achieved after 70 min at 180 °C.

ILs and DESs are often promoted as “green” but the veracity of this claim is unclear.^176^ Low vapor pressure, recyclability, metal-free nature, low toxicity, and biodegradability are given as proof of sustainability. Assessing the environmental impacts of a process is more complex than considering physical characteristics alone. For example, ionic liquid synthesis often requires significant organic-solvent use. A review from Jesus and Filho found that most ILs currently used are toxic, poorly-to-non-biodegradable, and more expensive than organic-solvent counterparts.^188^ Another analysis found phosphonium-based DESs display a toxic and cytotoxic effect greater than their individual component’s toxicity.^189^ Although this finding is not a complete picture of toxicity, it is an indication that ILs and DESs may not be as benign as reported.

Despite the explosion of papers involving ILs, there is a lack of high-quality LCAs to justify their use. A critical review by Maciel et al. pointed out that performed LCAs have found ILs/DESs have significant environmental impacts.^190^ As far as we are aware, no LCAs have been performed specifically on the use of ILs or DESs as solvents or catalysts for depolymerization. Zaib et al. performed an LCA on a (ChCl)/urea DES, known as reline, which showed better performance across seven environmental-impact factors than ethyl acetate and across four than DCM.^191^ However, ILs performed notably worse across all seven impact factors than ethanol or methanol. Sustainability claims, therefore, have to be specific. DESs have the potential to be worse than traditional solvents and more LCA is certainly needed in order to avoid unintended consequences.

### 2.6 Systemic Considerations: PET

PET is currently one of the most mechanically recycled plastics globally, with a recycling rate of around 50% in Europe in 2022.^192^ Mechanical recycling is cost-effective and significantly reduces environmental impacts. Unlike most polymers, mechanically recycled PET can achieve food-grade standards due to the homogeneity of PET recyclate (i.e., plastic bottles)^18^ and can achieve good recyclate quality due to the ability to rebuild molecular weight during polymerization. Depolymerization may have higher environmental impacts, but it produces higher quality products, making comparison complex.^193^ The relevant benefits of depolymerization needs to be targeted. These include removal of color, odor, and additives.^34^ Other key capability differences include depolymerization of PET from mixed plastic wastes and, importantly, from blends or multimaterials such as textiles or complex products. For example, although depolymerization of PET and other polyester fibers is possible by glycolysis, hydrolysis, and aminolysis, only 1% of the 180,000 tonnes of textile waste generated in Europe in 2016 were recycled into new clothes.^194^

Few depolymerization publications investigate mixed-postconsumer waste or textile waste as feedstocks. Thereby developing catalytic depolymerizations robust enough to deal with postconsumer-textile waste or which contain adequate pretreatment steps to facilitate this are of interest. For example, Huang et al. used a DBU catalyst to depolymerize PET-G (glycol-modified PET) from multilayer, multimaterial plastic payment cards.^195^ PET-G and metal contained within the payment cards were reclaimed at 1 kg scale, capturing value from a poorly recycled waste stream. A follow-on LCA and TEA for their DBU PET-G depolymerization found solving environmental hotspots like high water-consumption during BHET purification and reclaiming organic solvents came with increased economic cost and so pushed the minimum selling price up.^196^ More research like this with multimaterial and real-world waste are needed to uncover unforeseen trade-offs like this and so allow systems to be designed to solve them optimally, depending on prioritization (economic vs environmental).

Thermochemical recycling of PET is not an environmentally advantageous fate, so emphasis on developing depolymerization as a complement to mechanical recycling of PET is key,^45^ especially for multimaterials and copolymers that may help undercut the low price of virgin PET.

## 3 Polycarbonates

**Scheme 9:**
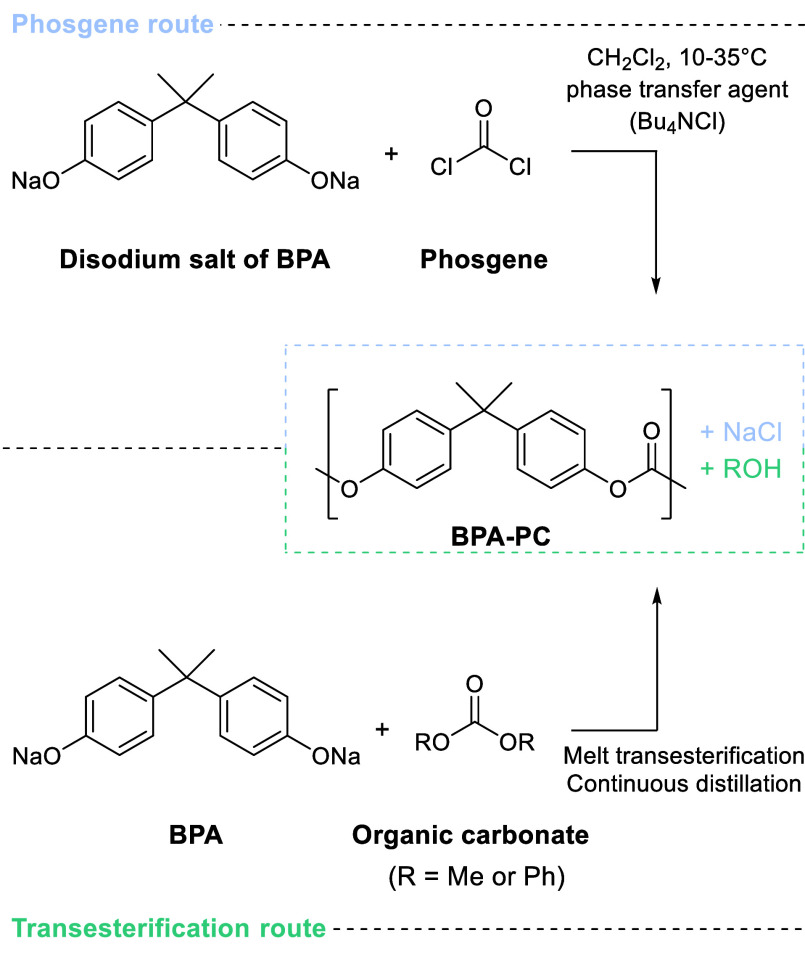
Two Main Commercial Synthesis Routes of BPA-PC Adapted with permission from ref (198). Copyright 2017 Elsevier.

Polycarbonates (PCs) are polymers with monomer units joined by carbonate linkages. Global consumption of PCs is significant, estimated at 5 million tonnes in 2022, with the vast majority being bisphenol-A polycarbonate (BPA-PC).^197^ BPA-PC is an amorphous engineering plastic prized for its transparency, high impact-strength, toughness, rigidity, and thermal stability (*T*_g_ 140–150 °C). BPA-PC has a range of applications including automotive parts and food and drink packaging. Two commercial BPA-PC production methods exist (Scheme 9): polycondensation of bisphenol-A (BPA) with phosgene or melt polycondensation of BPA with diphenyl carbonate.^198^ In both cases, BPA production is a key driver of environmentally detrimental impacts.^199^

The motivation to recycle BPA-PC is clear: to reduce environmental release of bisphenol A. Significant evidence indicates that bisphenol A is toxic and of environmental concern. Across many organisms, it has been shown to have thyroid-and-endocrine-disrupting, toxic, mutagenic, and carcinogenic effects.^200,201^ Currently, BPA-PC is poorly recycled by any method. Mechanical recycling is possible but hindered by a lack of collected PC waste in sufficient quantities. Sorting systems which rely on near-infrared (NIR) detectors for separation would likely sort PCs together with polyester waste, i.e., PET.

Chemically, the depolymerization of a polycarbonate is similar to that of a polyester. The carbonyl group in the carbonate is a target for nucleophilic attack. This attack can then be followed by liberation of CO_2_ or cyclic-carbonate formation depending on the chemistry used. BPA-PC typically degrades under milder conditions than PET because of the amorphous nature of BPA-PC. Furthermore, the extrusion of CO_2_ is an entropic driver for depolymerization and makes it effectively irreversible.

### 3.1 Bisphenol-A Polycarbonate

Approaches for BPA-PC depolymerization mainly comprise solvolyses (methanolysis, aminolysis, alcoholysis, hydrolysis). Methanolysis is most commonly reported in the literature, potentially due to the enhanced nucleophilicity of methanol, which affords milder reaction conditions. Methanolysis of BPA-PC with a TBD organocatalyst can yield 95% BPA after 2 h at 75 °C.^202^

Depending on temperature, methanolysis is heterogeneous, and the rate of BPA-PC swelling is important along with other kinetic drivers (temperature, catalyst etc.). Bhogle et al. performed NaOH-catalyzed methanolysis at 30 °C. Use of ultrasound enhanced the rate of swelling by inducing solvent cavitation.^203^ In another report, D’anna et al. described a mild methanolysis using ultrasound at 30 °C, with a [choline]_3_[PO_4_] catalyst. A 68% BPA yield was obtained after 105 min and was repeatable over 5 cycles.^204^ Liu et al. published a succinimide/DBU system as solvent and catalyst for the degradation of BPA-PC.^205^ A 96% BPA yield was obtained after 2 h at 70 °C. Trade-offs can be made between catalyst activity and robustness. Payne et al. demonstrated a Zn-Salen catalyst for the depolymerization of PET, BPA-PC, and poly(propylene carbonate).^206^ Remarkably >85% BPA yield was obtained after 2 h at 25 °C. The catalyst was air-sensitive so depolymerization occurred only under argon. Inert atmosphere requirement is commercially unattractive as the associated costs are substantial.

**Scheme 10:**
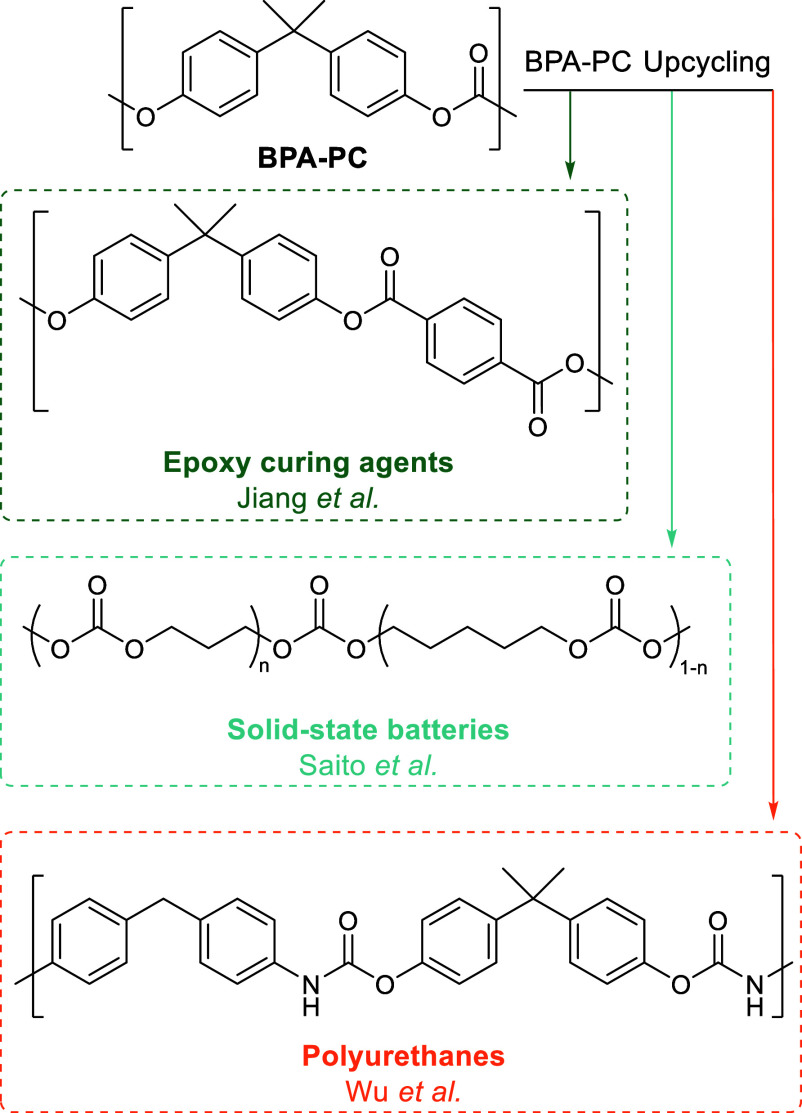
Examples of “Upcycling” Products from BPA-PC Depolymerization Adapted with permission from refs (207,208,210). Copyright 2020 MDPI, 2022 and 2018 American Chemical Society.

Interest in “upcycling” is present across the literature (Scheme 10). Saito et al. “upcycled” BPA-PC using TBD-MSA at 160 °C under N_2_ via carbonate containing diols to yield solid-polymer electrolytes for batteries.^207^ Jiang et al. used KOH-and-phase-transfer-agent-catalyzed basic hydrolysis of waste CDs (BPA-PC) to produce epoxide curing agents.^208^ Epoxy resins have also been prepared from BPA-PC by reaction with the epoxide DGEBA.^209^ Wu et al. depolymerized BPA-PC using aminolysis at 80 °C into BPA and carbamates, which were then repolymerized back into polyurethanes by addition of isocyanate.^210^ Accompanying LCA or TEA evidence of the preferentiality of the “upcycling” method is lacking across reported examples.

**Scheme 11:**
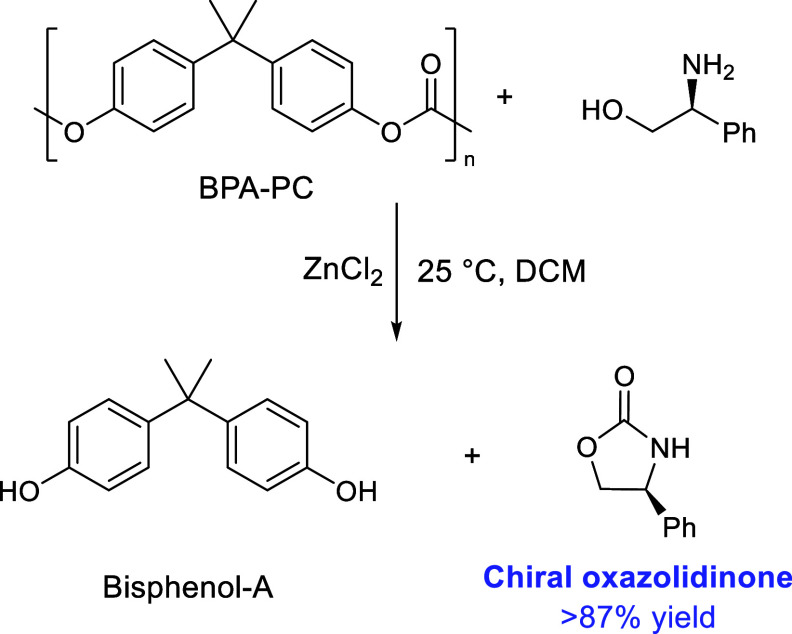
Formation of a Chiral Oxazolidinone from BPA-PC Using a Chiral Amino-Alcohol Adapted with permission from ref (213). Copyright 2022 American Chemical Society.

BPA-PC blends like polycarbonate/acrylonitrile butadiene styrene (PC/ABS) combine individual polymer-property advantages and have applications in electronics and automotives.^211^ As waste, PC/ABS would be suited to depolymerization. Sequential depolymerization of BPA-PC among other polyesters (e.g., PET, poly(lactic acid) (PLA) and polycaprolactone (PCL)) is gaining research interest. For example, Yang et al. reported a Zn(HMDS)_2_ catalyst which could depolymerize BPA-PC but not PET at 70 °C, and then increasing the temperature to 110 °C depolymerized the PET present.^212^ Wang et al. also obtained near-quantitative yields of BPA using a ZnCl_2_ catalyst at room temperature and then sequentially depolymerized PET.^213^ Furthermore, by including a chiral amino-alcohol as the nucleophile, good chiral-oxazolidinone yields (>87%) were reached as the product of BPA-PC depolymerization at 25 °C (Scheme 11).

Arias et al. tested a KOH-in-methanol hydrolysis process on a mixed PC/PET waste stream and achieved ∼85% BPA yield across a range of conditions by microwave heating to 120 °C in 2 min at 0.5 g scale.^214^ Jehanno et al. used TBD-MSA on BPA-PC/PET and achieved sequential depolymerization by increasing temperature from 130 °C up to 180 °C. After holding at 130 °C, all BPA-PC was depolymerized. Some PET had depolymerized (<5%), showing sequential depolymerization was achieved by a kinetic difference, with the rate of PET depolymerization being very low at 130 °C. Interestingly, a 1,3-diol was used as the solvent for the PC to form cyclic carbonates and BPA in good yields (88%) after 3 h. Furthermore, when applied to a commercial BPA-PC/PET blend (used in automotive body panels), 98% conversion of BPA-PC was reached in 6 h and only 4% PET was depolymerized.^215^ Jehanno et al. also used TBD-MSA to “upcycle” BPA-PC to BPA and a range of 5- and 6-membered rings. By using a 1,3-diol, diamine, or dithiol, different heterocycles were produced. The *gem*-dimethyl effect drove the formation of cyclic products, where unsubstituted 1,3-propanediol yielded only linear carbonates.^216^

### 3.2 Other Polycarbonates

New polycarbonates can be designed with structures conducive to chemical depolymerization. The design rationale of these novel polymers is discussed in section 7. The two main chemical approaches for designing depolymerization into novel polycarbonates take advantage of either CO_2_ liberation with cyclic epoxide formation, or *trans*-cyclic carbonate formation.^25,217^ For example, Yu et al. produced a suite of pyrrolidine centered polycarbonates by polymerizing *N*-heteroepoxides with CO_2_.^218^ Varying alkyl substituents on the 5-membered ring afforded control over the thermal properties and a *T*_g_ similar to BPA-PC was obtained.

Polycarbonate synthesis from cyclic epoxides and CO_2_ is attractive for producing both linear and cyclic polymer architectures.^219^ Informative reviews provide a deeper look at the catalyst systems employed.^220,221^ While not comprehensive, it is useful to highlight some specific avenues for bioderived polycarbonates, including vanillin-derived imine carbonates^222^ and limonene/limonene-oxide-derived polycarbonates.^223^ Siragusa et al. synthesized polycarbonates from biomass-derived diols and bicyclic carbonates.^224^ Pendent ketone inclusion and use of aminolysis meant room temperature depolymerization was achieved in just 30 min with no catalyst. Saxon et al. synthesized a range of functionalized polycarbonates from bioderived alcohols, with tunable thermal properties that depended on the functional group.^225^ Chemical depolymerization was demonstrated using DBU at 90 °C for 24 h and yielded the cyclic-carbonate-functionalized alcohol (ROH) and CO_2_.

**Figure 8:**
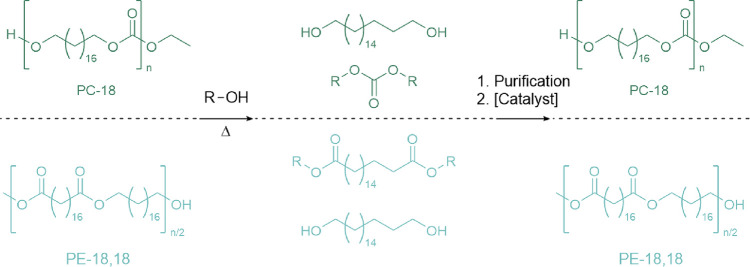
Depolymerization of the polyethylene-like polycarbonates (PC-18) and polyesters (PE-18,18). The depolymerization catalyst used was 10 wt % KOH, relative to polymer mass. Adapted with permission from ref (226). Copyright 2021 Nature.

Mecking and colleagues prepared polycarbonates and polyesters with a low density of carbonate/ester functional groups from long-chain C_18_ oleates (Figure 8).^226^ Inclusion of long-chain oleates afforded crystallinity and chain arrangement similar to that of HDPE, with resultant HDPE-like mechanical properties (elongation at break of 350% and 470% and Young’s modulus of 640 and 910 MPa for the polycarbonate and polyester, respectively), while the low density of carbonates added chemical targets for triggered depolymerization. Hydrolysis, catalyzed by 10 wt % KOH in EtOH at 120 °C, afforded quantitative monomer recovery after 1 h. Depolymerization was also shown by KOH-catalyzed hydrolysis in water at 180 °C. Consideration of end-of-life fates concluded that by sink–float or NIR-based spectroscopic sorting techniques, the HDPE-like polymer would remain in a polyolefin recycling waste stream. Separation of the polycarbonates from the polyolefins HDPE, PP, and LDPE was possible by polycarbonate depolymerization, which left the polyolefins unaffected. However, implementation of this sorting-by-depolymerization at mechanical reprocessing plants is unlikely due to cost, complexity, and the negligible amounts of polyethylene-like carbonates to be found in general postconsumer-plastic waste streams.

Abe et al. depolymerized poly(isosorbide carbonate) by ammonolysis at 90 °C for 6 h to yield isosorbide and urea which are used as fertilizer.^227^*Arabidopsis* plants were fertilized with the isosorbide and urea, and more plant growth was observed. However, this conversion to fertilizer represents a suboptimal fate for the polymer, as the fertilizer does not retain high material-value and is single use. McGuire et al. performed a solid-state depolymerization of poly(cyclohexene carbonate) using a dinuclear Mg–Co catalyst with >99% selectivity for a range of cyclic epoxide products at 140 °C under N_2_ flow.^228^ Catalyst loading was as low as 0.01 mol %. Entropically favorable release of CO_2_ biased the depolymerization equilibrium toward monomer formation. Yu et al. pyrolyzed poly(cyclohexene carbonate) in the presence of a Cr^III^–Salen complex with a bis(triphenylphospine)iminium-based cocatalyst at 200 °C for 20 min under vacuum, and obtained >99% cyclohexene oxide conversion using only 0.2 mol % catalyst at a 5 g scale.^229^

Liu et al. reported the transfer hydrogenation of poly(propylene carbonate) and obtained 65% propylene glycol and 43% MeOH using an iron pincer catalyst at 140 °C after 30 h.^230^ Jiang and Thomas produced a polycarbonate from dimethyl carbonate and (1*R*,3*S*)-(+)-*cis*-1,2,2-trimethylcyclopentane-1,3-dimethanol (TCDM), a camphor-derived product.^231^ Methanolysis was achieved by a 1:20 MeOH to THF solvent mix after 72 h at 100 °C by a magnesium catalyst.

### 3.3 Systemic Considerations: Polycarbonates

Mechanical recycling is a physically possible but currently unrealized fate for polycarbonate waste. The challenging reprocessing conditions and the lack of established collection and sorting routes limit economic viability. Depolymerization may open new avenues to reclaim value, as it is more readily scaled down and can be used to treat mixed postconsumer wastes.^232^ Reports have demonstrated depolymerization of BPA-PC in the presence of PET and PLA, as they are often commingled by current waste-management systems (i.e., near-infrared sorting).^215^ However, many blends like PC/ABS contain depolymerization-resistant polymers, calling for innovative process design to recover more value than depolymerization alone. Sequential depolymerization appears attractive as it removes sorting steps. Conversely, a universal catalyst could depolymerize all three at once, with product separation rather than three sequential depolymerization processes. Developing an LCA and TEA database could assist in defining a preferred system, which could then help define future catalyst development.

The depolymerization methodology would also facilitate monomer recovery from other sectors. Polycarbonates are often applied in polymer blends, so depolymerizations that could reclaim monomers and value from these blends would add complementary capabilities compared to mechanical or thermochemical recycling. Polycarbonate blends have commercial applications in automotives, where collection by manufacturers and recycling may be more feasible, as collection feasibility can be sector specific.^233^ Shifting from BPA-PC to reduce bisphenol A release; it is important that catalysts and systems will accommodate new BPA-free PCs as they emerge on the market. A potential challenge may be the plurality of new PCs, further complicating collection and separation.

## 4 Polyamides

**Figure 9:**
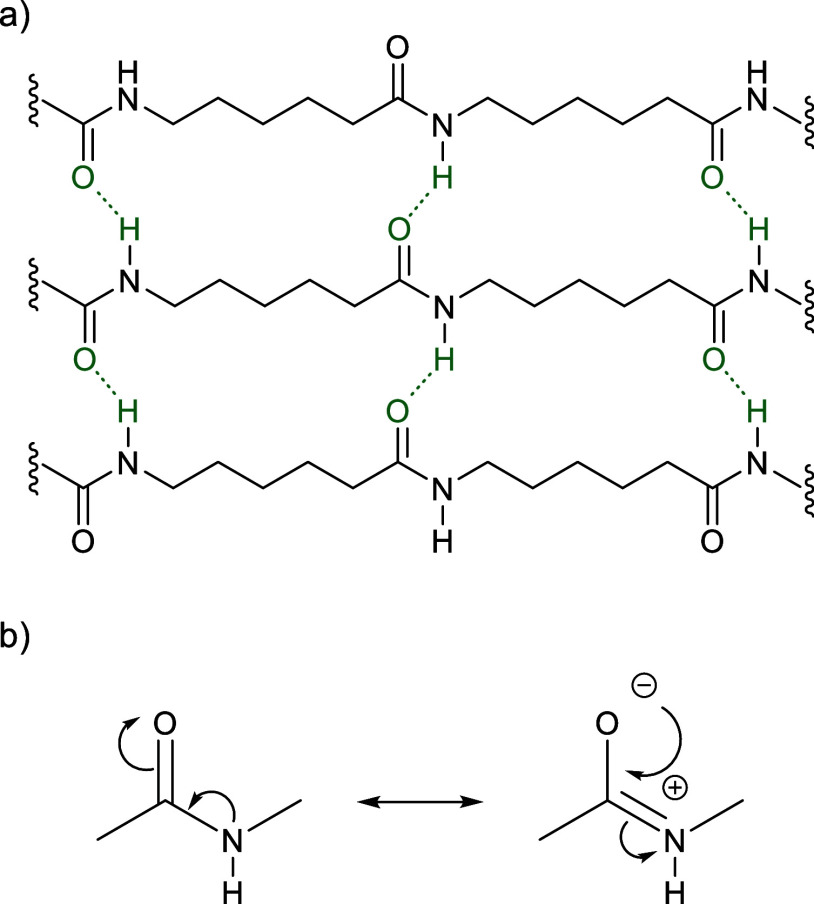
(a) Intermolecular hydrogen bonding in nylon-6, which increases both mechanical strength and crystallinity. (b) Resonance stabilization of an amide bond, which makes it more resistant to nucleophilic attack.

Polyamides are a versatile class of polymer applied as textiles, fishing nets, high-performance sports equipment, and composite reinforcements. Among PAs, nylon-6 and nylon-66 are the most widely used. The term nylon refers specifically to aliphatic polyamides. Amide bonds endow polymer chains with strong intermolecular hydrogen bonding, making polyamides mechanically robust (e.g., tensile strength at break = 50–95 MPa, elongation at break = 150–300%, and Young’s Modulus = 0.8–3.5 GPa),^234^ especially under tension (Figure 9a). PAs are often semicrystalline and have good thermal and chemical stability. Amides can be degraded through acid/base-catalyzed hydrolysis or solvolysis. When compared to polyesters, depolymerization of polyamides requires harsher conditions because of enhanced conjugation between the carbonyl and nitrogen atom (Figure 9b).

PA wastes are often multimaterials which pose a challenge for recycling. Polyamide fibers such as from nylon carpet waste are unsuitable for mechanical recycling, as fibers require bespoke recycling processes.^14^ Mechanical recycling is also hindered by the inclusion of other components (namely polypropylene and fillers like CaCO_3_) in high proportion. Another challenge for the recycling of polyamides is effective sorting of nylon-6 from nylon-66. Zagar et al. demonstrated determination of nylon-6 and nylon-66 content of a mixed waste stream of the two by HCl-catalyzed hydrolysis and then using a refractive-index-detector-equipped HPLC.^235^ Carpet waste was found to contain 57 wt % nylon-6, with the rest being styrene butadiene latex (13%) and 29% of the flame-retardant Al(OH)_3_. Fishing nets were 96% nylon-6 and 2–3% bitumen, used as a hydrophobic coating.

### 4.1 Nylon-6

**Figure 10:**
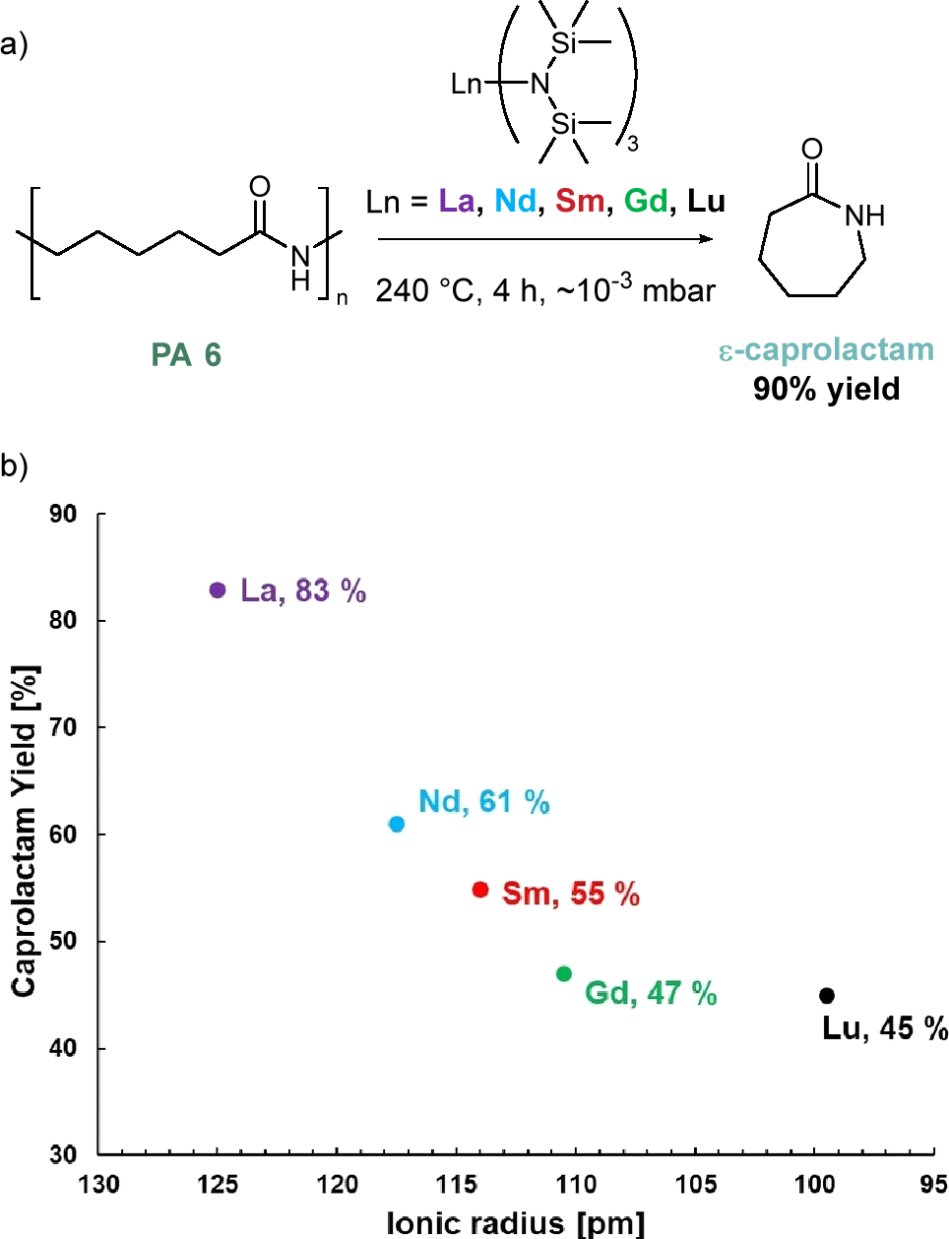
(a) The lanthanide catalyst system used by Wursthorn et al. to achieve nylon-6 depolymerization. (b) Graph of the dependence of yield on the ionic radius of the lanthanide metal used in the catalyst. (a) Adapted and (b) reproduced with permission from ref (237). Copyright 2023 John Wiley & Sons.

Nylon-6 (PA 6) is made by ring-opening polymerization of ε-caprolactam. Ring-closing depolymerization is the primary degradation pathway under elevated temperatures, yielding ε-caprolactam.^236^ However, the ring-closing process requires temperatures of 350–500 °C. By adding a catalyst, this nylon pyrolysis can be done at lower temperatures (240 °C) and was recently shown to achieve >95% selectivity for ε-caprolactam and excellent yields (85–90%) (Figure 10a,b).^59,237^ A series of lanthanide catalysts were reported, with steric clashing and crowding hypothesized as leading to lower yields at smaller ionic lanthanide radii. Furthermore, supercritical steam with a range of catalysts have also been explored to this end.^238^

Chemical depolymerizations of nylon-6 to ε-caprolactam can be milder than heating alone. Alberti et al. depolymerized nylon-6 using 4-dimethylaminopyridine and acetic anhydride at 260 °C over 15 min.^239^ A 73% yield of the N-acetylated caprolactam was obtained, with other linear side products. When applied to postconsumer samples, 20–78% yields were observed depending on the nylon-6 source. To obtain ε-caprolactam, trans-acetylation by ethanolamine provided a 92% yield after 2 h at 80 °C. As PA wastes are often multimaterials, innovative solutions are required to recover value. Chen and Bai reported a combined hydropulping, THF/acid treatment, solubility separation, and finally pyrolysis of PA to yield ε-caprolactam from food and drink carton waste.^240^

### 4.2 Nylon-66

Nylon-66 is made by polycondensation of adipic acid and hexamethylenediamine. The structure and applications of nylon-66 and nylon-6 are similar, however nylon-66 has stronger interchain hydrogen bonding and increased crystallinity.^241^ This bonding makes nylon-66 more mechanically, thermally, and chemically robust than nylon-6.

**Scheme 12:**
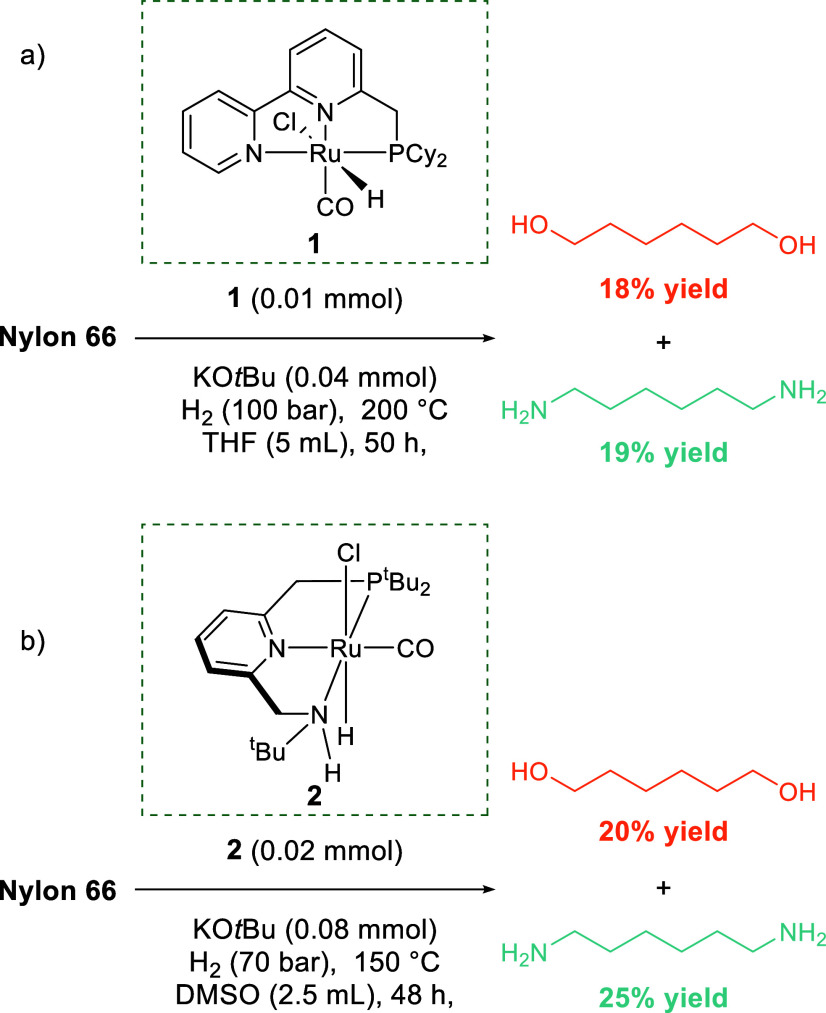
(a) Hydrogenolysis Reported by Zhou et al.; (b) Hydrogenolysis Reported by Kumar et al. Adapted with permission from refs (243) and (242). Copyright 2021 Wiley-VCh and 2020 American Chemical Society.

Hydrogenolysis of nylon-66 (and nylon-6) with a ruthenium-pincer catalyst was recently and simultaneously reported by two groups. Kumar et al. performed hydrogenolysis in DMSO with a Ru catalyst and were able to depolymerize nylon-6, nylon-66, and a range of other PAs (Scheme 12b).^242^ Yields were boosted by sequential catalyst addition, suggesting catalyst deactivation was present. Zhou et al. performed hydrogenolysis of nylon-66 and Ultramid A27, a commercial-grade PA (Scheme 12a).^243^ Optimized conditions of 0.01 mmol of ruthenium-pincer catalyst to 0.5 g of Ultramid A27 and 0.04 mmol of a KO*t*Bu cocatalyst, all under 100 bar of H_2_, in 5 mL of THF at 200 °C for 50 h, yielded 19% and 18% of diamine and diol, respectively. Solubility of the nylon in THF limited yields. Together, these hydrogenolysis reports are an interesting proof-of-concept of a new avenue for PA depolymerization. However, yields and selectivity for monomer over dimer/trimer require great improvement if the approaches are to have commercial potential and even then, end markets capable of accepting the amine/alcohol products from nylon-6 waste are poorly defined.

Datta et al. depolymerized nylon-66 by glycolysis and used the product to replace polyols in polyurethane synthesis.^244^ To date, there are only a few reported examples of nylon-66 depolymerizations, potentially suggesting low commercial pull from industry for non-circular targets.

### 4.3 Other Polyamides

Aromatic polyamides, or aramids, include commercially relevant polymers such as Kevlar. The combination of stiff aromatic rings, π–π stacking between chains, and a high density of amide bonds makes them extremely robust to chemical degradation. The solubility of aramids in organic solvents is poor, ruling out some traditional solvolysis methods. Instead, sub- and supercritical water have been used to depolymerize them at elevated temperature (250 °C) and pressure (4 MPa).^245^ Supercritical water hydrolysis can lead to substrate carbonization and poor monomer yields. By adding NaOH, depolymerization in subcritical water at lower temperatures can be done, with shorter reactions times. However, stoichiometric base is consumed, and salt waste generated, making the process costly and inefficient.

Cesarek et al. depolymerized nylon-66, -1010, -11, and -12 in the presence of glass or carbon fiber reinforcement fibers.^246^ Acid hydrolysis was utilized with a 1.25:1 HCl-to-amide molar ratio for nylon-66 at 200 °C (via microwave heating) over 10 min. HCl was catalytic but also consumed in the protonation of the formed amine groups. Carbon fiber reinforcements fibers were recovered in good condition and the monomers obtained were of very high purity after recrystallization (>99%).

### 4.4 Systemic Considerations: Polyamides

Mechanical recycling is possible for engineering polyamides, particularly of off-cuts from products. This may increase with legislative levers such as extended producer responsibility.^247^ Textiles, a major proportion of nylon-6 wastes, are difficult to mechanically recycle, while thicker woven fibers in fishing-gear recycling have had limited success. While nylon-6 is thermochemically convertible back to monomer in near-quantitative yields, applications are hindered by the heterogeneous nature of nylon-6 waste and challenging collection environments. The sorting of nylon-6 from nylon-66 is another potential challenge, as collection would likely first cosegregate all polyamides. With two potential recovery paths, a second separation step may be needed, adding to economic and environmental burdens. Pretreatments steps are necessary to provide suitably pure and contaminant-free polyamides for depolymerization, as these are a major barrier to closed-loop recycling.^248^ For example, commercial recycling of nylon-6 from old fishing nets to consumer products, including skateboards, presently occurs and involves an intensive pretreatment step to ensure recyclate quality.^249^ There is a lack of LCA on nylon depolymerization approaches, and in particular, consideration of depolymerization of mixed wastes by LCA.^193^ While LCAs on commercial systems are known, peer-reviewed assessment of these is needed. Many textiles contain both polyamides and polyesters (i.e., PET), and so exploration of depolymerizing both of these together would be a key barrier to overcome. For example, during Peng et al.’s investigation of PET/nylon-6-textile acetolysis, both polymers depolymerized with TPA purification unaffected, while the ethylene glycol diacetate and 6-acetylaminocaproic acid products were separated by distillation.^104^ Therefore, the challenges of mixed textile waste can be overcome but with increased process complexity and environmental cost.

## 5 Aliphatic Polyesters

Aliphatic polyesters have received extensive academic and industrial interest. The aliphatic ester moiety is more susceptible to hydrolysis under environmental conditions, although many systems are fully biodegradable under industrial composting conditions. This same biodegradability enabler also installs a chemically depolymerizable functionality in these polyesters, and so research has also investigated their chemical depolymerization.

### 5.1 Poly(lactic acid)

Poly(lactic acid) (PLA) is an aliphatic polyester made up of repeat units of lactic acid. PLA is a renewable plastic as lactic acid and lactide can both be derived from fermentation of plant sugars. Lactic acid is chiral, with two enantiomeric forms (d-lactide and l-lactide). Racemic mixtures of the two, polymerized with nonstereospecific catalysts yield amorphous PLA (PDLLA), while PLA grades rich in either enantiomer (PDLA and PLLA) are semicrystalline. Most commercial PLA sold today is PLLA or PDLLA due to the natural occurrence of l-lactide, although recent catalyst innovations mean PDLA is also available, but at higher cost. PLA is thermoplastic with a low melting point (140–180 °C) and mechanical properties including brittleness (for racemic PDLLA, elongation at break = 2.5–6%, tensile strength = 21–60 MPa, and Young’s Modulus = 0.35–3.5 GPa) but otherwise similar to polystyrene and PET.^250^ PLA is more susceptible to hydrolytic and thermal degradation than PET, with degradation occurring at temperatures over 200 °C. Commercially, approximately 400,000 tonnes of PLA were produced in 2022, and its main applications include disposable packaging, medical implants, drug delivery systems, automotive components, and textiles.^250,251^

PLA has two main production routes. One is by the polycondensation of lactic acid, with water as a side-product. The second way PLA is made is by the ring-opening polymerization of lactide using a metal catalyst, commonly tin octanoate. PLA is known for being biodegradable, however, it is only biodegradable in industrial composters or digestors, which operate at raised temperatures (>60 °C). PLA does not significantly break down if landfilled, or home composted, with degradation taking years, similar to “non-biodegradable” plastics. Therefore, the main potential recycling routes for PLA are mechanical or chemical. PLA is mechanically recyclable, however, low thermal and hydrolytic stability mean significant property deterioration occurs after even one recycle.^252^

Reported PLA depolymerization methods include hydrolysis and aminolysis. PLA is not water-soluble, and addition of organic cosolvents can be beneficial. The activation energy of hydrolysis has been reported as 9% lower in a 50:50 water ethanol mixture than in pure water at moderate temperature (60–90 °C).^253^ Shao et al. depolymerized PLLA with ethanolamine to obtain the amide *N*-lactoyl ethanolamine, which was then converted into a dimethacrylate ester for use as a 3D-amylprinting photocurable resin.^254^ The effect of temperature, nucleophile:PLLA (feed) ratio and time were investigated. The significance of parameters in descending order was: temperature > time > feed ratio.

**Scheme 13:**
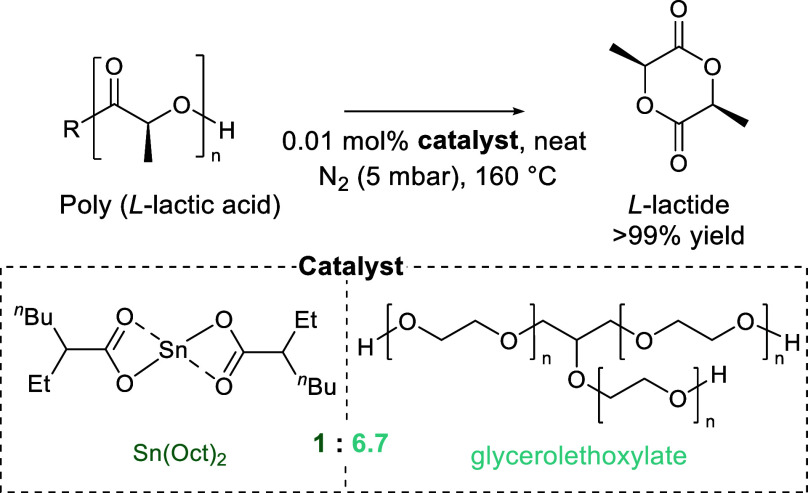
Sn(Oct)_2_ Catalyst Used by McGuire et al. for Stereo-Controlled Depolymerization of PLLA Adapted from ref (257). Copyright 2023 American Chemical Society.

Wang and colleagues have investigated Zn(HMDS)_2_ as an efficient catalyst to depolymerize PLLA, producing methyl lactate in excellent yields (92%) at 25 °C.^255^ Their system was able to selectively depolymerize PLLA at room temperature in one-pot mixtures of PLLA/PBAT and PLLA/PBS, with the other polymer depolymerizing by raising the temperature to 50 °C.^212^ Further, postconsumer PLA cups and straws were depolymerized and, with addition of l-lactic acid, repolymerized with good molecular weight achieved (*M*_n_ = 35.5–71.6 kg/mol).^256^ Depolymerization took 6 h at 25 °C in DCM. McGuire et al. depolymerized poly(l-lactic acid) (PLLA) to l-lactide in over 99% yield in a stereocontrolled depolymerization, which took advantage of a Sn(Oct)_2_ catalyst enhanced by a triol additive and sublimation of formed lactide to preclude detrimental further side reactions (Scheme 13).^257^

Another promising route for PLA depolymerization is use of alcohol nucleophiles to generate alkyl lactates. Majgaonkar et al. synthesized ethyl lactate from a range of virgin and postconsumer PLAs by using a TBD catalyst, ethanol, and acetone solvent at 50 °C.^258^ 3 equivalents of ethanol to 1 equivalent of PLA repeat unit afforded a good (80%) yield after 1 h. Increasing the amount of ethanol actually slowed the depolymerization rate by hindering dissolution of the PLA. Nim et al. used a range of diols (ethylene glycol, 1,3-propanediol, 1,4-butanediol) and a tetrabutyl orthotitanate catalyst to synthesize a mix of lactate, lactyllactate, and polylactates. These were then used as polyols to make PU films.^259^ Zinc catalysts have also been widely investigated for the production of alkyl lactates with a conversion of 97% after 1 h at 50 °C.^260^ English et al. used N-heterocyclic phosphines as catalysts for the methanolysis of PLA. A >70% methyl lactate yield was obtainable after 60 h at 80 °C in toluene.^261^ Nunes et al. used a molybdenum catalyst to depolymerize PLA from 3D printer waste, using silane in toluene at 110 °C to obtain propane. A 95–100% conversion was achieved after 20 or 40 h. The PLA scale, however, was small (0.5 mmol of PLA).^262^

LCA evidence suggests that all recycling treatments of PLA are better than incineration or composting in terms of environmental impacts.^263^ Piemonte and Gironi suggest that chemical depolymerization of PLA by hydrolysis is preferable to virgin production energetically. Mechanical recycling was found as preferable to chemical recycling in terms of energy, human health, ecosystem quality, and resource consumption.^264^

**Figure 11:**
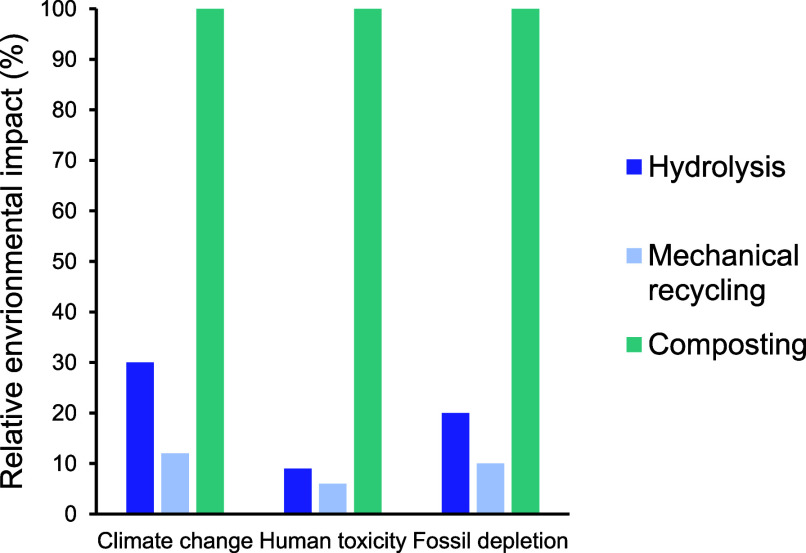
Relative environmental impacts of hydrolysis, mechanical recycling, and composting of waste PLA. Adapted with permission from ref (16). Copyright 2016 Springer.

De Andrade et al. performed a comparative LCA of the PLA end-of-life fates: mechanical recycling, chemical recycling by hydrolysis, and composting (Figure 11). Mechanical recycling was assumed to produce PLA of virgin quality, based on successful use of chain extenders. Hydrolysis was assumed to be acatalytic at 180 °C for 2 h, using 40 wt % PLA in water. To obtain a 98% LA yield, water distillation and impurity removal were needed. The findings across climate change, human toxicity, and fossil depletion were: composting was the worst with 100% value, chemical recycling second best with 10–30% value, and mechanical recycling had 5–12% value using the restitution system.^16^ For hydrolysis, electricity consumption drives impacts with 44% being the hydrolysis and 50% being the repolymerization.

Aryan et al. compared the impacts of hydrolysis, methanolysis, ethanolysis, and incineration.^265^ All three depolymerization technologies were found to be better than incineration. Hydrolysis performed best on land usage. Ethanolysis was better from a global warming perspective when compared to methanolysis, potentially due to increased yield, although it performed worse in other metrics. Ethanol further benefitted as methanolysis relies on fossil fuel derived methanol where ethanol can be biosourced, leading to biogenic carbon storage. Further, ethyl lactate has many more potential solvents it can substitute for than methyl lactate. However, alcoholysis and hydrolysis cannot be directly compared as they produce different products. The downstream processing (i.e., separation, purification) of products in hydrolysis was a major driver of negative impact factors, including global warming potential.

### 5.2 Polycaprolactone

Polycaprolactone is an aliphatic polyester, commercially made by the ring-opening polymerization of ε-caprolactone with a tin octanoate catalyst.^266^ PCL has found uses in medical implants, sutures, and targeted drug release.^267^ Another important and larger-scale application of PCL is in the production of thermoplastic polyurethanes. At end of life, PCL, like PLA, has many possible fates. PCL films have been shown to biodegrade over hundreds of days in 25 °C compost.^268^ Greater value still could be recaptured by depolymerization, with examples reported of hydrolysis, methanolysis, and thermally driven ring-closing depolymerization.^269−272^ Mechanical recycling of PCL has received little academic or commercial interest, perhaps due to negligible amounts of available PCL waste in a feasibly recyclable form. Academically, some other interesting treatments of PCL waste explored include hydrosilylation and enzymatic degradation by lipases.^273,274^

Another important waste stream is the depolymerization of PCL blends, and PCL-based thermoplastic polyurethanes. PCL/thermoplastic polyurethane (TPU) blends can access interesting property profiles with features like shape memory or self-healing ability.^275,276^ PCL is also used as the “soft segment” in TPU production to obtain biocompatible materials for medical applications.^277^ In both cases, mechanical recycling would be a poor outcome, as the mixed plastics produce heterogeneous blends with unsuitable mechanical properties. PCL is unlikely to become a widely used polymer in the near-future, and a lack of adequate collection precludes commercial depolymerization.

### 5.3 Polyhydroxyalkanoates

Polyhydroxyalkanoates (PHAs) are a class of biodegradable polyester, including examples like polyhydroxybutyrate (PHB) and polyhydroxyvalerate. PHAs occur naturally and are made by a range of microorganisms through fermentation of sugars or lipids. PHAs share a common structure, but around 150 different examples have been reported depending on alkyl chain length between ester groups and alkyl substituents.^278^ PHA’s properties vary depending on the exact structure but are similar to polypropylene, and as such PHAs have been used as packaging materials, medical implants, sutures, and other biocompatible parts.

**Scheme 14:**
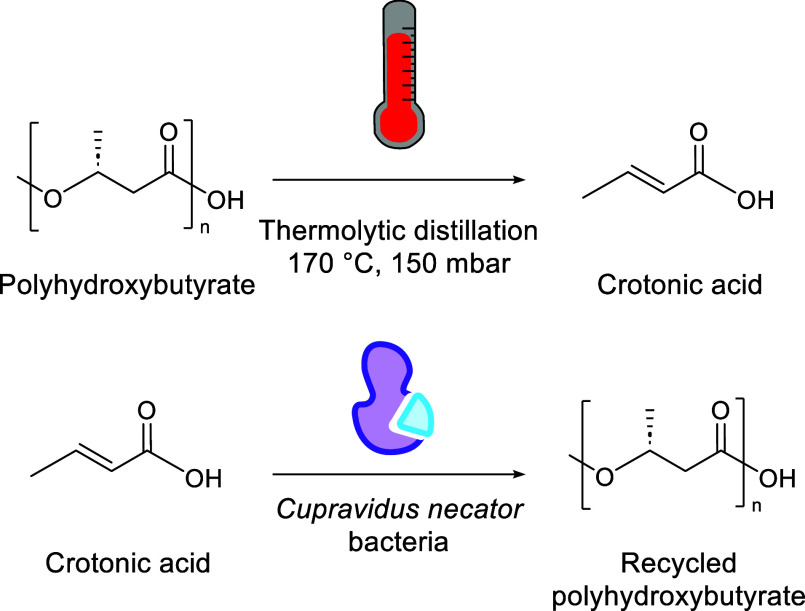
Thermolytic Distillation and PHB-to-PHB Process Developed by Parodi et al. Adapted from ref (281). Copyright 2022 Elsevier.

Parodi et al. exploited a novel thermolytic distillation process through which PHB is distilled at 170 °C and 150 mbar, which led to a thermolysis of the ester and desaturation to produce crotonic acid in excellent yields (92 wt %) using pure PHB substrate.^279^ The process was more energy efficient than similar thermal depolymerizations of PHB. In a later work, the process was elaborated upon as a key step in producing the biosolvent methyl 3-methoxybutyrate from PHB.^280^ In a further contribution, the same thermolysis process was applied to PHBs to produce CA which was then fed to *Cupriavidus necator* bacteria for fermentation into more PHB (Scheme 14).^281^ An overall, PHB-to-PHB yield of 55% for the tandem process was achieved. The energy performance was modeled and found as lower than alternatives by 20–25%.

Methyl 3-methoxybutyrate is a common chemical target for the depolymerization of PHB in particular. It can be accessed via methanolysis, with ILs being reported as good catalysts.^282,283^ Lehnertz et al. explored ruthenium on CeO_2_ support as a heterogeneous catalyst for conversion of PHB to 3-hydroxybutyric acid under H_2_.^284^ Wang et al. reported on (Zn(HMDS)_2_) as a transesterification catalyst for PHB with ethanol and *n*-butanol in bulk reactions to the corresponding esters.^285^ The tacticity of the PHB was an important factor in determining the rate as atactic PHB reached 94.3% conversion in 2 h at 110 °C, whereas isotactic only reached 90.3% conversion in 3 h under the same conditions.

As polyesters, PHAs are susceptible to similar solvolysis as those discussed for PET and PC. Hydrolysis in acidic or basic media of PHAs has been explored, however, recent publications are scarce.^286^ Far more recent publications have explored enzymatic hydrolysis of PHAs, although further discussion is beyond the scope of this review.^287^ Examples of novel, synthetically produced PHAs with altered and non-traditional structures are a current research focus, aiming to maximize chemical recyclability and bridge failings like lack of melt-reprocessability and mechanical failure.^288^

### 5.4 Poly(butylene adipate terephthalate)

Poly(butylene adipate terephthalate) (PBAT) is a biodegradable, fossil-derived, polyester that is a random copolymer of adipic acid, 1,4-butanediol, and terephthalic acid. PBAT is used in agricultural mulch films, biodegradable plastic bags, and cling wrap for food. At end of life, biodegradation of PBAT is slow; when buried in soil, one study found 2.3% mass loss after 3 months.^289^

**Scheme 15:**
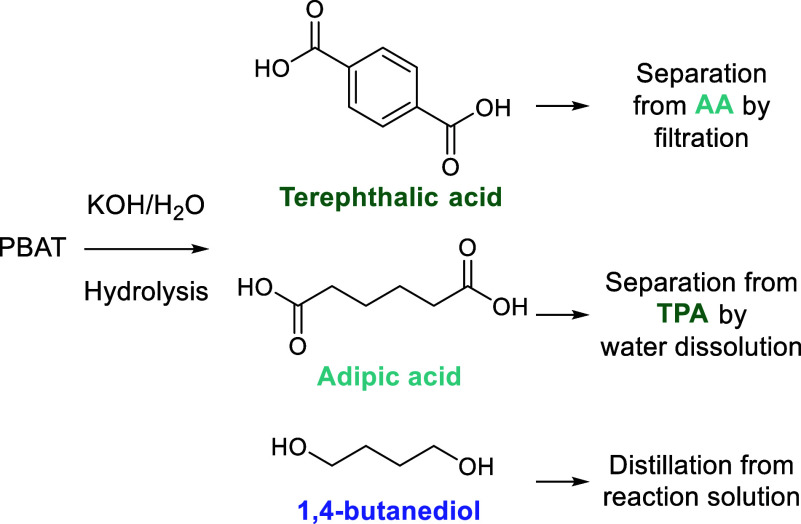
Products of PBAT Hydrolysis Followed by Separation Method Adapted with permission from ref (290). Copyright 2023 American Chemical Society.

In terms of depolymerization, PBAT is susceptible to degradation conditions similar to other polyesters. For example, Yang et al. depolymerized PBAT by methanolysis with a Zn(HMDS)_2_ catalyst and obtained a good yield (89%) of DMT after 9 h at 100 °C.^212^ PBAT was more resistant to methanolysis than PLA, which depolymerized at room temperature in just 2 h to give 98% methyl lactic acid. Alkaline hydrolysis is also a promising technique for the depolymerization of PBAT. Shen et al. used a KOH in an ethanol/water mix and obtained excellent yields after 50 min at 80 °C.^290^ A challenge peculiar to PBAT was noted: separation and isolation of three products. By precipitating TPA and adipic acid out as potassium salts, the normal use of acids to neutralize them was reduced by ∼10%. In turn, the differing water solubility of TPA and adipic acid were leveraged to allow separation (Scheme 15).

There is a dearth of LCAs investigating the chemical, mechanical, and biological recycling of PBAT. Without such data, it is difficult to compare the impacts of chemical recycling of PBAT vs enzymatic vs biodegradation in the natural environment. Shen et al. considered the carbon footprint of their process and found it had lower carbon emissions than virgin production.

### 5.5 Systemic Considerations: Aliphatic Polyesters

Despite concerted academic and commercial interest, aliphatic polyesters remain a small part of the mix of commercial plastics used today. While the end of life for biodegradable polymers is more diverse, most still require a systemic management. As many of these polymers can be mechanically, chemically, or biologically processed at end of life, the design of future systems is even more important for these less established materials. Renewability or biodegradability are often poorly contextualized in terms of environmental sustainability; more specific LCA can help evidence such claims.^291^ The small scale of biodegradable plastic use and their ready degradation similarly makes mechanical recycling challenging. Depolymerization could thus be particularly apt at creating value from these streams. Coupling biomass/CO_2_ uptake to produce polyesters and depolymerization to keep this CO_2_ in the cycle could enable genuine net-zero plastics.^39^

The removal of these products for chemical recycling can also improve mechanical recycling systems. As PLA contamination severely impacts PET mechanical recycling of PET, it may be a priority for chemical depolyermization collection.^291^ In the long term, producing bioplastics from bioresources and depolymerizing to circularize them at end of life seems favorable, with PHAs in particular holding promise to replace polyolefins.^292^ However, to reach this vision, we again highlight the need for quantifying environmental impact: land use for producing plastic vs food, their effects on mechanical recycling, a lack of dedicated collection/sorting, and the need to view the polymers as complementary parts of our plastics system.

## 6 Polyurethanes

**Scheme 16:**
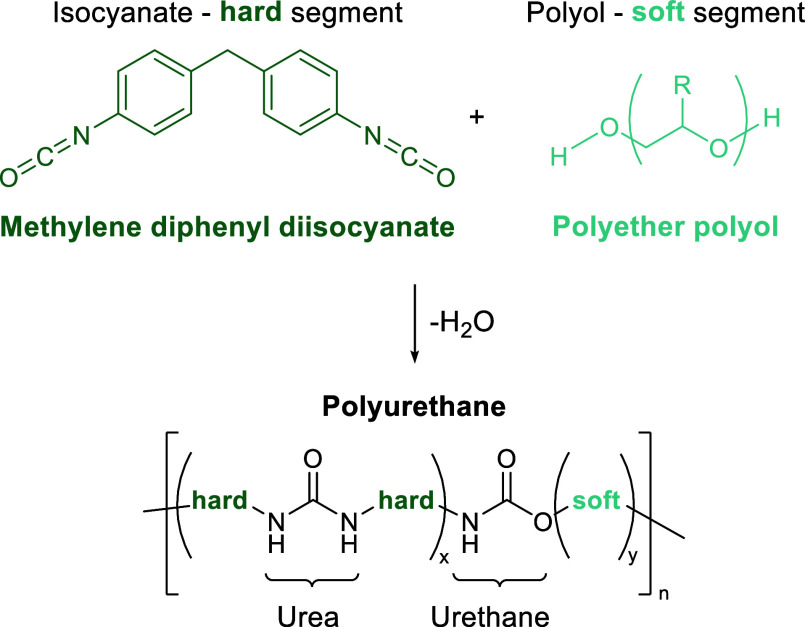
General Synthesis of Polyurethanes Isocyanate sections are “hard” because of their highly ordered chains with strong interchain interactions (hydrogen bonding and π–π interactions).

Polyurethanes are a significant (18.6 MMT global demand in 2021)^24^ and often overlooked class of polymer waste. Chemically, PUs are constituted by urethane linkages between monomer sections. PU synthesis is typically achieved via condensation of a difunctional aromatic isocyanate and a polyol alcohol, with concomitant liberation of water (Scheme 16). Within a PU, aromatic isocyanates act as “hard” rigid segments, and polyols act as counterbalancing “soft”, flexible sections. By varying the stoichiometry and identity of these two key components, a wide array of property profiles are accessible.^293^ PUs can be thermoplastics or thermosets and have applications as foams, coatings, elastomers, fibers, and adhesives. As a result, PU waste streams can contain a mixture of PU forms, complicating recycling. Thermoset PU foams are 73% of the global PU market as of 2017 and are a significant proportion of PU waste.^293^ PU recycling is further complicated by the many different isocyanate and polyols, cross-linkers, and other additives used in product formulations. PU depolymerization plants often produce recycled polyols with substandard properties that require treatment, adding to costs and environmental impacts.^294^

Depolymerization by glycolysis, aminolysis, and hydrolysis have all been reported.^295−297^ Glycolysis is industrially applied on massive scale, a rare example of commercial success for depolymerization methods. Glycolysis is predicted to remain an underpinning technology of broader PU recycling.^298^

### 6.1 Thermoset Polyurethanes

Thermoset PUs are the majority of PUs produced globally and are a challenge for depolymerization. Recovery of isocyanates via glycolysis is not feasible as glycol-capped products are yielded by transesterification. Instead, polyol recovery is simpler and more widely reported. Inclusion of recycled polyols can improve the compressive strength, thermal insulation, and self-extinguishing properties of PUs.^299^

**Figure 12:**
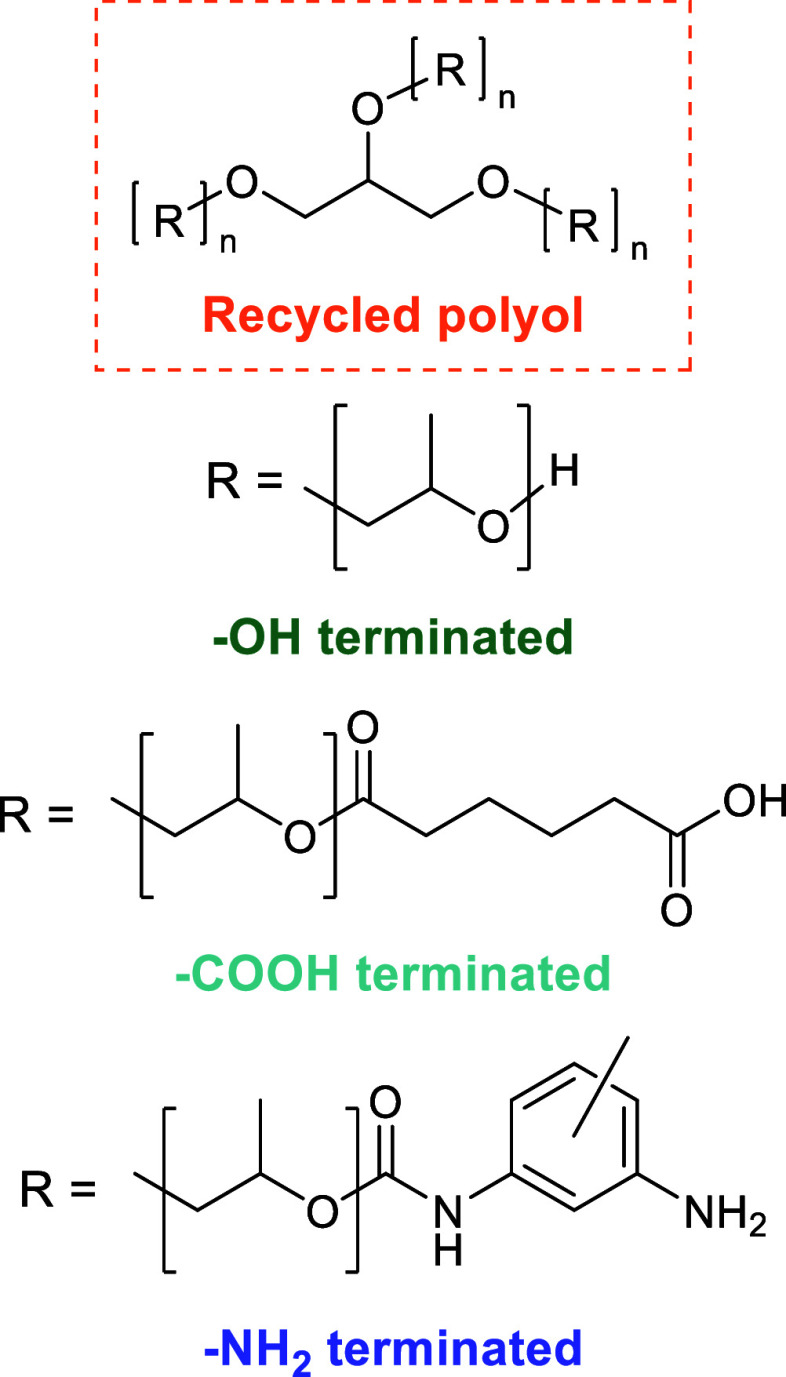
Different polyol end-groups obtained by Grdadolnik et al. through competing side reactions during adipic acid PU acidolysis. Adapted from ref (301). Copyright 2022 American Chemical Society.

Acidolysis is a promising method for polyol recycling from waste PU foams. When applied to fiber-reinforced PU composites, acidolysis with HCl has been applied to recover carbon fibers intact.^300^ Acidolysis with dicarboxylic acids often takes advantage of a polyol solvent to separate the hard and soft segments by their differing solubility. Urethane linkages are transesterified and converted to amide bonds, resulting in carboxylic acid terminated hard segments and polyols. Grdadolnik et al. investigated the acidolysis of virgin and postconsumer PU foam with adipic acid.^301^ The reaction used microwave heating to 210–230 °C for 15–40 min. Varying temperature and duration dramatically impacted the proportion of −COOH, −NH_2_, and toluenediamine obtained in the recycled polyols (Figure 12). This proportion in turn impacted the properties of a PU foam made with recycled polyols. Particularly, increased time and temperature lead to a higher degree of urethane conversion, but also esterification of the polyol end-groups with adipic acid, yielding a −COOH end group instead. Synthesis of new PU foam with 0–100% recycled polyol content significantly impacted the unit cell size and subsequently density (virgin = 27.1 kg m^–3^ vs 100% recycled polyol = 29.7 kg m^–3^) and mechanical properties (compressive modulus of virgin = 18.1 kPa vs 100% recycled polyol = 26.2 kPa) of the foams produced. Other dicarboxylic acids including succinic and phthalic have been investigated for PU foam acidolysis.^302−304^ To circumvent the consequences of the competing esterification and transesterification side reactions, the applicability of recycled polyols in PU coating and adhesives has been investigated.^303,304^ Overall, acidolysis processes can rapidly depolymerize PU foams but many products and side products are formed, compromising the quality and amount of recycled polyol obtained.

Johansen and colleagues investigated *tert*-amyl alcohol as a solvent/catalyst for PU depolymerization. With addition of base (<1 wt % KOH) or MeOH, postconsumer flexible and rigid foams and solids were depolymerized over 2.25–6.00 h at 225 °C. High yields of toluenediamine (>95%), a diisocyanate precursor, and polyols were recovered.^305^ In a further work, they showed that the recycled polyol could 100% substitute virgin polyol in PU synthesis, while maintaining good mechanical performance (e.g., virgin PU tensile strength = 93 kPa, and recycled PU tensile strength = 96 kPa).^306^ Zhao and Semetey investigated the methanolysis of PUs using a range of basic catalysts (DBU, TBD, *tert*-butoxide).^307^ Under mild conditions (65 °C, 20 h), good yields (≤80%) of the methanol-terminated transcarbamoylation product were obtained. This product was repolymerized into PU, reducing use of environmentally harmful isocyanates in PU production. Motokucho et al. performed a high pressure (≤16.1 MPa), CO_2_-assisted hydrolysis process to recover diols and diamines from PUs.^308^ Under these conditions, competing esterification and other side reactions were disfavored by the lack of catalyst, solvent, and lower temperature used. He et al. combined an initial ammonolysis with a subsequent acidolysis, taking advantage of the increased reactivity of ammonolysis for milder conditions followed by acidolysis to yield suitably terminated polyols.^309^

Dynamic polyurethane networks have been reported as a novel PU material type with enhanced chemical recyclability.^310^ Huang et al. prepared dynamically cross-linked thiourethanes and demonstrated their facile depolymerization. The mild depolymerization (4% TMG, 9 equivalents of thiol monomer, 25 °C, 4 h) of these thiourethanes shows their promise to circumvent the use of problematic isocyanates. A string of recent publications have detailed the hydrogenolysis of PU by transition metal catalysts: Ir, Ru, and Mn.^242,243,311^ As an exemplar, Gausas et al. achieved depolymerization of both synthesized and postconsumer polyurethanes, from different important PU waste types: flexible foams and solids and their rigid counterparts. Under optimum conditions, 86% anilene yield was achieved using a commercial iridium-pincer catalyst at 30 bar H_2_ and 150 °C. Anilenes were recovered from postconsumer samples, but yields were significantly lower.

### 6.2 Thermoplastic Polyurethanes

Thermoplastic polyurethanes are so-called because of their meltability. TPUs are prepared by reaction extrusion of diisocyanates, diols, chain extenders, and other additives. TPUs have applications as wiring insulation, footwear, medical devices, and phone cases, to name a few. TPUs can be depolymerized like thermoset PUs, and their lack of cross-linking facilitates quicker depolymerization. Yan et al. prepared TPUs from δ-caprolactone and MDI, which were depolymerized using Sn(Oct)_2_ at 180 °C over 2 h.^312^ δ-Caprolactone was produced in near-quantitative yield (99%), but no isocyanate was recovered. TPUs have received significantly less attention for depolymerization than thermoset PUs despite similar underlying chemistry.

**Figure 13:**
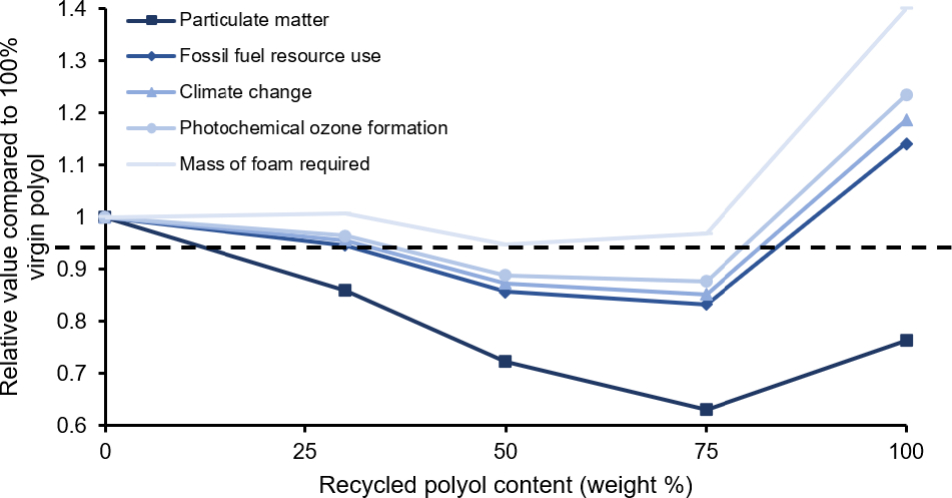
Graph of the relative environmental impacts of including recycled polyol content in PUs from 0 to 100%. Adapted with permission from ref (313). Copyright 2021 American Chemical Society.

There is a lack of reported LCAs on PU depolymerization, considering PUs are the sixth most produced commercial polymer globally.^24^ A recent analysis on the benefits of substituting virgin polyols with those from glycolysis was informative.^313^ Incorporation of recycled polyols, up to 75% inclusion, significantly reduced environmental impacts. For example, impacts were reduced by ∼25% in the climate change impact category (Figure 13). A 100% recycled polyol inclusion was actually detrimental as poorer physical properties were observed, thus demanding a greater amount of material for the same purpose, negating the benefit from recycling. A perspective piece on the area pointed out that while transcarbamoylation and hydrogenolysis approaches are promising, and benefits can be obtained from finding lower energy and use of green solvents, there is a lack of evidence to support their environmental preferability.^314^

### 6.3 Systemic Considerations: Polyurethanes

Polyurethane depolymerization is a success story, with industrial-scale glycolysis of foams and rigids producing recycled polyols, showcasing how industrial synergies can help overcome chemical recycling technologies at low technological readiness levels.^315^ The quality of this success story needs to be underpinned by LCA of PU depolymerizations. Extending product recovery to chemicals other than polyols may be key to improving TEAs. Other catalytic depolymerization chemistries than glycolysis need developing to this end. Reactive isocyanates are hard to recover, and the diversity of PU formulations precludes large-scale depolymerization processes producing pure products.

This is especially true for PU thermosets, where mechanical grinding and compounding is not truly circular and thermochemical approaches are confounded by the high heteroatom content of Pus. Depolymerization remains the most technically feasible option for PU recycling in a circular plastic economy. This may be tied to improved polymer design, where isocyanate-free syntheses for PUs could open repolymerizable target products on depolymerization.^232^ Separation of complex products and high energy input are key barriers for PU depolymerization,^294^ which again highlights the importance of simplification and standardization of formulation. In this way, design for end of life would enable depolymerization, and a circularization of PU waste.

## 7 Designer Polymers

Commercial polymers were originally developed to valorize waste generated in energy production, prioritizing economics over environment. Designing for end of life and chemical depolymerization is thus a much more recent target. This concept has come to be embodied in the literature by the term “chemical recycling to monomer” (CRM).^25^ The guiding principle in controlling depolymerization is balancing the thermodynamics and kinetics of polymerization vs depolymerization (see Coates et al. for deeper discussion).^25^ During polymerization, monomers and polymers are in equilibrium. The position of this equilibrium is mainly governed by the chemical structure of a given monomer/polymer; catalysts do not alter the position of thermodynamic equilibrium but rather alter the pathways between polymer and monomer.

The Gibbs free energy of polymerization (Δ*G*_p_) determines the spontaneity of polymerization. If Δ*G*_p_ < 0, polymerization occurs spontaneously. If Δ*G*_p_ > 0, depolymerization occurs spontaneously. For polymerizations, when Δ*G*_p_ is equal to zero, the system is at equilibrium and the amount of polymer and monomer remains constant. The temperature at which this occurs is typically called the ceiling temperature (*T*_c_). For designer chemical structures, temperature and dilution are key variables by which the position of equilibrium can be altered. CRM seeks to choose monomer/polymer systems with a moderate *T*_c_. Too high, and the depolymerization process will be energetically costly, and unwanted side reactions can occur (e.g., thermolysis of polyethylene). Too low, and the polymer may depolymerize during processing or service life.

### 7.1 Lactones

The ring-opening polymerization of a range of lactones has been exploited to design depolymerizable polymers. Many examples using γ-butyrolactone (γ-BL) and its derivatives have been published, prominently by E. Y. X. Chen and colleagues. Their first report investigated a range of catalysts, notably including La[N(SiMe_3_)_2_]_3_, for γ-BL polymerization, achieving high monomer conversion (>90%), molecular weight (*M*_n_ up to 30.2 kDa), and acceptable dispersity control.^316^ Crucially, near-quantitative monomer recovery was attained by heating at 220 °C for 1 h. Further works explored a range of polymerization and depolymerization catalysts, as well as different γ-BL derivatives to achieve well controlled, high-molecular-weight polymers with depolymerization possible by catalysts including *tert*-Bu-P_4_,^317^ ZnCl_2_,^318−320^ and TBD^321^ , under mild temperatures (40–120 °C). Chen and colleagues also depicted the preparation of a copolymer of γ-BL and poly(glycolic acid), and its corresponding depolymerization under basic aqueous conditions.^322^

**Scheme 17:**
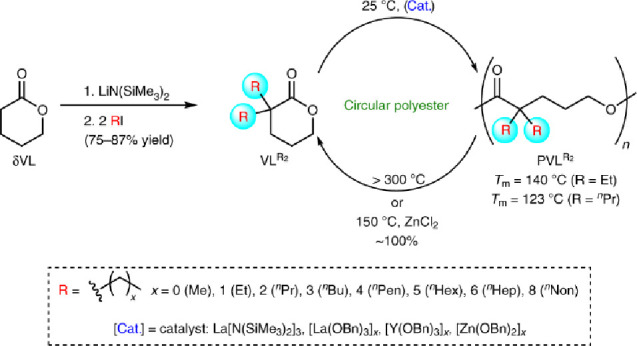
δ-Valerolactone CRM System Developed by E. Y. X. Chen and Colleagues with Conversion to a Circular Polyester Reproduced with permission from ref (323). Copyright 2023 Springer Nature BV.

Valerolactones^323−325^ (Scheme 17) and δ-decalactones^326,327^ are notable lactones which have been explored as constituents of polymers with facile chemical recycling to monomer. These polymers combined mild depolymerizability with mechanical and barrier properties comparable to commercial polymers. For example, poly(δ-valerolactone) at *M*_n_ = 66.1 kg mol^–1^ had a Young’s Modulus of 493 MPa, an elongation at break of 904% and a tensile strength at break of 66.6 MPa compared to 190 MPa, 385%, and 12 MPa, respectively, for an LDPE comparator.^325^

### 7.2 Nontraditional Polyhydroxyalkanoates

PHAs, as previously discussed, are an important class of biosourced polyester with tunable and attractive properties. Zhou et al. reported on chemically recyclable PHAs with excellent mechanical and thermal properties.^288^ By introducing α,α-substitution with methyl groups (α to the carbonyl), the properties of the PHAs were significantly enhanced and the depolymerization was favored by overcoming ring strain via the *gem*-dimethyl effect of the dimethyl substituents. Depolymerization was achieved by hydrolysis using aqueous LiOH in THF/MeOH at 100 °C over 72 h, under glovebox conditions. A similar work described the synthesis of poly(3-hydroxy-2-methylbutyrate)s from 2,3-dimethyl-β-propiolactone monomers. Depolymerization in the presence of 1 wt % MgO at 200 °C yielded 93% tiglic acid. Although not the parent monomer, it was pointed out that tiglic acid can be used as a feedstock for biosynthesis of poly(3-hydroxy-2-methylbutyrate).^328^

### 7.3 Other Polymer Types

A range of novel polymers of differing type have been investigated in the search for excellent physical properties combined with simple, controllable, and mild depolymerization processes. Gregory et al. prepared block copolymers based on trimethylene carbonate and vinyl-cyclohexene oxide or limonene oxide with impressive mechanical strength (tensile strength = 60 MPa) and elasticity (>800% extension with 95% recovery), which behaved like thermoplastic elastomers.^329^ Depolymerization was possible using TBD catalyst over 3 h at 80 °C.

In an exciting development, Abel et al. produced high molecular weight (*M*_n_ 220 kDa) poly(1,3-dioxolane) (PDXL) via reversible-deactivation cationic ring-opening polymerization.^330^ The PDXL displayed mechanical properties (tensile strength at break = 40 MPa, elongation at break = 720%) comparable to commercial polymers LDPE, HDPE, and *i*PP along with good thermal (*T*_d,50%_ > 380 °C in the presence of weak acids and amines) and hydrolytic stability (no hydrolytic degradation observed even when dissolved in water above its ceiling temperature). The corresponding depolymerization gave near-quantitative yields of monomer via distillation at 140 °C in ambient air, using a camphorsulfonic acid catalyst. An alternate depolymerization catalyzed by a commercial acidic resin (Dowex-50) at 150 °C gave near-quantitative yields after 1 h at ∼15 g scale. Key to attaining CRM was the low ceiling temperature of the polymer (*T*_c_ = 13 °C). Depolymerization in the presence of mixed plastic waste was selective for PDXL. In a later elaboration, Hester et al. switched the system to an oxonium salt catalyst and produced ultra-high-molecular-weight PDXL (*M*_n_ > 2000 kDa) while maintaining chemical recyclability under the same conditions with 93% monomer yield.^331^

Carrodeguas et al. prepared CO_2_, limonene oxide, and ε-decalactone block copolymers with molecular weights of up to *M*_n_ = 114.6 kDa and tunable mechanical properties by varying block size.^332^ Depolymerization was facile in toluene with a dizinc catalyst at 80 °C to recover limonene oxide and CO_2_. Polydecalactone was further depolymerizable by addition of PTSA. Block copolymer polyester/carbonates have been widely depolymerized and have tunable properties.^333^

Other efforts have explored chemically recyclable thermosets. These approaches take advantage of different bonds/monomer chemistries with highlights including imines, boroxines, diketoenamines, and polyethers.^334−338^ Some included dynamic networks which paired the potential for thermal remolding and recovery of properties with full depolymerizability. For more on dynamic networks and their recycling, see ref (339).

**Scheme 18:**
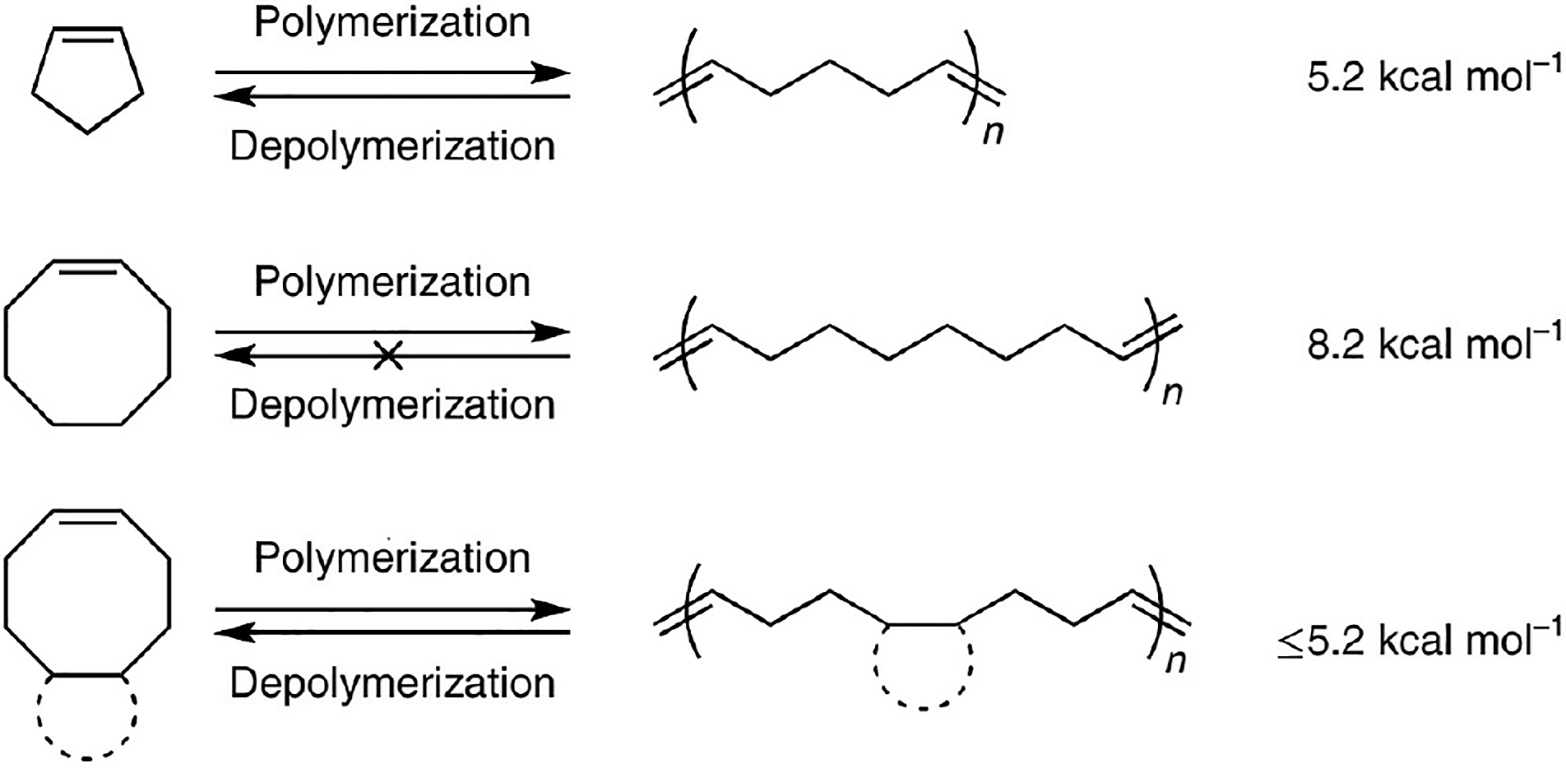
Sathe et al. Included a Second Fused Ring (Bottom) to Increase the Ring Strain Energy, Shown on the Right for Each System, of Cyclooctene Based Polymers Reproduced with permission from ref (340). Copyright 2023 Springer Nature BV.

Sathe et al. reported the ring-opening metathesis polymerization of a bicyclic cyclooctene (Scheme 18).^340^ Inclusion of a second (*trans*-cyclobutane) fused ring induced increased ring strain, thus favoring depolymerization by ring-closing metathesis. Depolymerization occurred in CHCl_3_ with 1 mol % of second-generation Grubb’s catalyst at 50 °C after 2 h with >90% monomer recovery. Later work investigated the effect of other fused rings, including cyclic acetals. Geminal substituents on these cyclic acetals were found to reduce the *T*_c_ and thus enabling CRM.^341^

**Figure 14:**
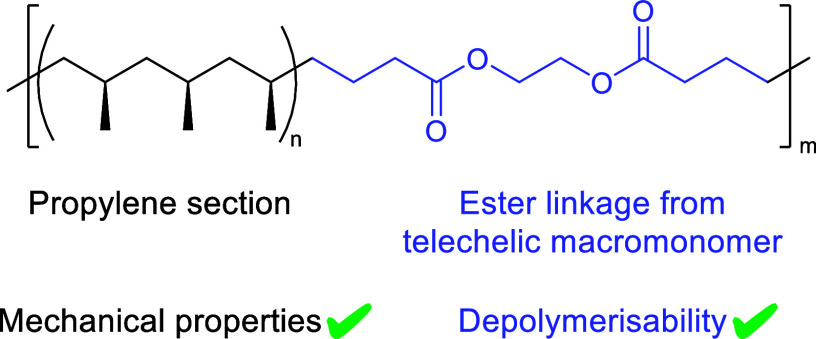
Propylene-based polymer prepared by Kocen et al. to incorporate both tunable mechanical properties with depolymerizability. Adapted with permission from ref (343). Copyright 2022 American Chemical Society.

Cywar et al. produced a nylon-4/6 hybrid of ε-caprolactam and pyrrolidone to combine their advantages; nylon-6 shows good thermal and mechanical properties but a high *T*_c_, whereas nylon-4 exhibits the opposite.^342^ Depolymerization by pyrolysis was carried out at 290 °C under optimal conditions with a ZnCl_2_ catalyst, which yielded 93–98% mass recovery after 15–18 h. Undoubtedly, catalyst improvements would be necessary for broader application as such a long and energy intensive process is industrially unattractive. Kocen et al. developed a butadiene propylene copolymer with macromonomer segments linked by ester groups.^343^ This copolymerization strategy endowed chemical depolymerization to recover these olefin macromonomer segments (Figure 14).

Efforts to design new polymers for chemical recyclability are encouraging to develop intrinsically more sustainable materials. Across the examples discussed, complementary follow-on LCA and/or TEA analysis of the depolymerization process would provide insight into their environmental preferability and feasibility for commercial application. In some cases, depolymerization was not notably milder or simpler than for commercial polymers. Indeed, this lack of preferable depolymerization showcases the difficult trade-offs made in developing polymers which are simple, controllably (de)polymerizable, and have excellent properties. A common challenge is that polymers designed for depolymerizability have lower stability; better end of life properties come at the cost of poorer service life properties.

### 7.4 Systemic Considerations: Novel Polymers

Designing for depolymerization is developing rapidly. The marriage between the readily polymerizable/depolymerizable early work and improved polymer properties, often by accessing higher molecular weights, has been key. Evaluating the position of these polymers in a broader plastics system is essential, necessitating LCA and TEA studies as well as the impact of these new polymers on existing waste management fates. For these new systems, the footprint of polymer production may outweigh potential end-of-life gains, as is the case in recent work on poly(mandelic acid).^344^

The barrier to improving LCA and TEA incorporation is rooted in interdisciplinarity, and we highlight the need to both standardize expectations and the upskilling of chemical science researchers to holistically consider end of life and service life through the lens of economic environmental and societal impacts.^345^ Many systems target selective depolymerization in the presence of other plastics (a mixed waste scenario), saving on sorting costs. These new polymers would necessitate a prospective depolymerization plant of sequential reactors depolymerize each waste, with associated changes in solvents, temperature, catalyst, and potentially atmosphere. Such a system needs significant attention, as avoiding one burden (sorting) can add another. As pointed out by Beckham and colleagues,^346^ depolymerization processes could operate three ways: by sorting plastic before depolymerization (potentially simpler than separating chemical products after), by sequential depolymerization as described, or by applying a universal catalyst which depolymerizes all plastics present, with products then either reacted together or chemically separated. Finding out which is optimal for different waste streams would be key for highlighting improvements needed by future chemical recycling to monomer research.

It is also important to note that designer polymers are not limited to designer fates. These new polymers may be more easily mechanically recycled, which could be beneficial or harmful to existing mechanical recycling systems, as this is likely to remain the predominant recycling technology in the near future.^347^

## 8 Outlook and Conclusions

A transition to a circular plastics economy is essential to safeguard the natural environment, climate, and human condition. Mechanical, thermochemical, and depolymerization approaches can only enable this transition when viewed together rather than in isolation. Refining the role of depolymerization in this interconnected system of end-of-life fates can define economic opportunities that also reduce environmental harm. The technologies discussed in this review may prove pivotal to circularizing the current linear use model, however, technological focus alone will be insufficient to achieving a sustainable and circular plastics-use system. More focus is needed on how technologies, like depolymerization, are evaluated within these broader systemic constraints, be they economic, social, or environmental.

Across the polymer classes featured in this review, there has been substantial development over the last 5 years. Growing research attention to mixed, postconsumer, and more realistic waste streams is encouraging along with better application of environmental (LCA) and economic (TEA) analyses to guide progress. Novel polymer types show great promise, even at this early stage. Analyses like LCA and TEA need to be consistently applied to both established and emerging systems to define the most promising systems forward. This is especially true as fundamental chemistry interacts with the complexity of global waste management provision. Mechanical recycling remains environmentally and economically optimal for many current plastic waste streams, including many discussed in this review. Developing chemical recycling approaches to be complementary can underpin a more comprehensive and dramatic system change.

Waste streams suitable for this immediate complementarity include thermosets, multimaterials, polymer blends, and copolymers, all currently poorly served by existing recycling infrastructure. Chemical challenges include catalyst sensitivity to contaminated postconsumer waste, interaction with additives, and identification of end markets suitable for “upcycled” products. There are a scarce few examples of translation to higher technology readiness levels. Higher yielding, lower cost, and more energy efficient catalytic systems will no doubt help bridge this gap.

Commercial barriers should not stifle the exciting, exploratory research into new catalyst systems and polymer types designed for CRM. Overall, chemical depolymerization research would benefit from broader systemic considerations of how the investigated systems will fit with current plastic use, waste managements, and indeed, other recycling technologies. With this in hand, a transition to a sustainable, circular plastics system is a future we can strive for and look forward to.

## Abbreviations

ABS: acrylonitrile butadiene styrene
BHET: bis(2-hydroxyethyl) terephthalate)
BHETA: bis(2-hydroxy ethyl)terephthalamide
BPA: bisphenol-A
BPA-PC: bisphenol-A polycarbonate
CRM: chemical recycling to monomer
DBN: 1,5-diazabicyclo[4.3.0]non-5-ene
DBU: 1,8-diazabicyclo[5.4.0]undec-7-ene
DES: deep eutectic solvent
DFT: density functional theory
DMT: dimethyl terephthalate
EG: ethylene glycol
GHG: greenhouse gas
HDPE: high-density polyethylene
HMDS: bis(trimethylsilyl)amide
IL: ionic liquid
LCA: life cycle analysis
LDPE: low-density polyethylene
*M*_n_: number-average molecular weight
MSA: methanesulfonic acid
1,5-NDSA: 1,5-naphthalenedisulfonic acid
NIR: near-infrared
2-NSA: 2-naphthalenesulfonic acid
PA: polyamide
PBAT: poly(butylene adipate terephthalate)
PC: polycarbonate
PCL: polycaprolactone
PDXL: poly(1,3-dioxolane)
PET: poly(ethylene terephthalate)
PET-G: glycol-modified poly(ethylene terephthalate)
PHA: polyhydroxyalkanoate
PHB: polyhydroxybutyrate
PLA: poly(lactic acid)
PLLA: poly(l-lactic acid)
PP: polypropylene
PS: polystyrene
PTSA: *p*-toluenesulfonic acid
PU: polyurethane
TBD: 1,5,7-triazabicyclo[4.4.0]dec-5-ene
*T*_c_: ceiling temperature
TEA: techno-economic analysis
*T*_g_: glass transition temperature
TPA: terephthalic acid
TPU: thermoplastic polyurethane
γ-BL: γ-butyrolactone
