# Correction to Depolymerization within a Circular Plastics System

**DOI:** 10.1021/acs.chemrev.4c00473

**Published:** 2024-07-11

**Authors:** Robbie A. Clark, Michael P. Shaver

**Affiliations:** † Department of Materials, School of Natural Sciences, University of Manchester, Manchester M13 9PL, United Kingdom; ‡ Sustainable Materials Innovation Hub, Henry Royce Institute, University of Manchester, Manchester M13 9PL, United Kingdom

There is an error in a structure in Scheme 3. The corrected Scheme is provided here.

**Scheme 3:**
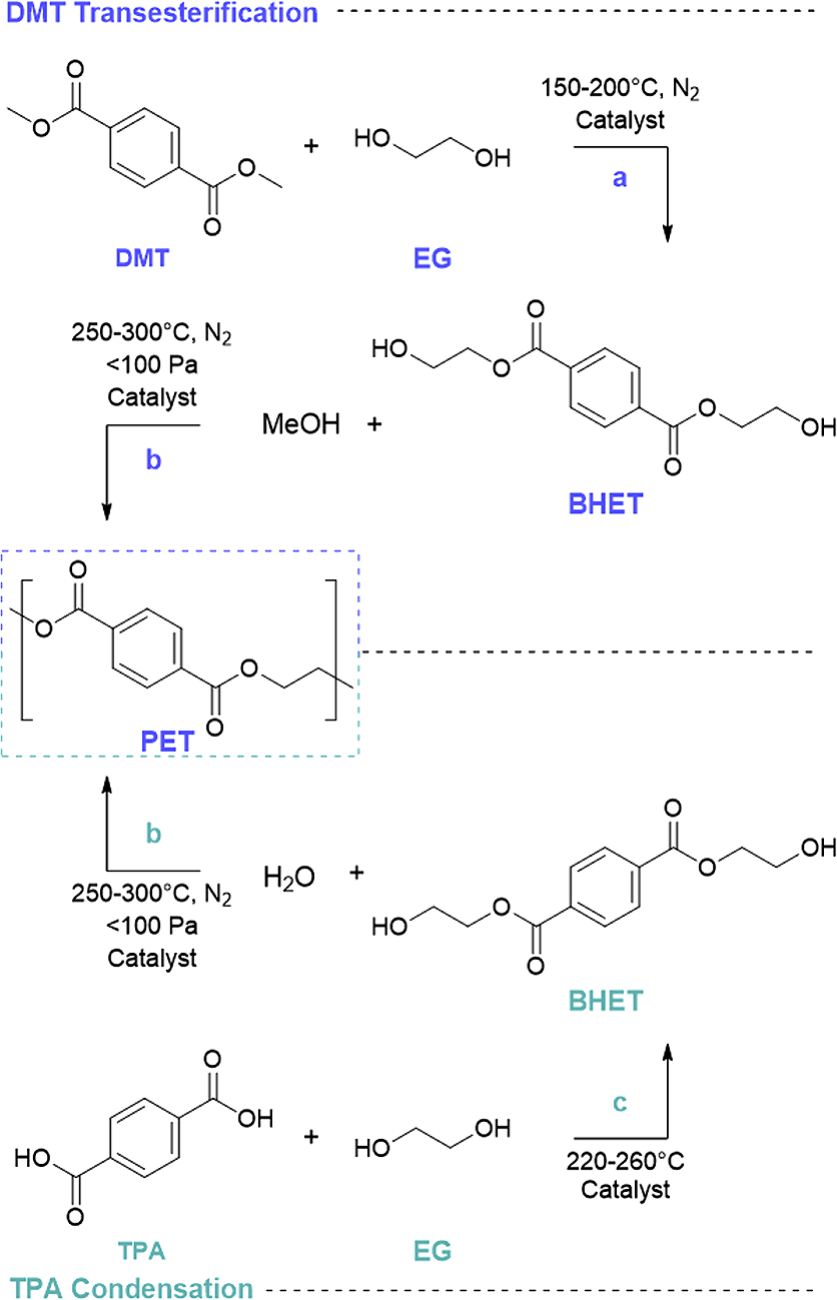
Production of PET by Two Main Commercial Methods. (a) Catalytic Transesterification and Methanol Formation by Amine, Metal Alkoxide and Metal Acetate Catalysts. (b) Polycondensation Using Sb, Ge, Ti, or Pb Catalysts at High Temperatures and Reduced Pressures. (c) Esterification of TPA and EG with a Transesterification Catalyst Adapted from ref 65.

There is also a typographical error in the title of Scheme 8. The new title reads:

**Scheme 8. Typical Methanolysis Conditions. Catalysts Include Bases, Lewis Acids, and Ionic Liquids**

